# An Enantiospecific Synthesis of 5-*epi*-α-Bulnesene

**DOI:** 10.3390/molecules28093900

**Published:** 2023-05-05

**Authors:** Jiarui Zong, Jeremy Robertson

**Affiliations:** 1 Chemistry Research Laboratory, Department of Chemistry, University of Oxford, Mansfield Road, Oxford OX1 3TA, UK; jiarui.zong@chem.ox.ac.uk; 2 Oxford Suzhou Centre for Advanced Research, Ruo Shui Road, Suzhou Industrial Park, Suzhou 215123, China

**Keywords:** agarwood, incense, fragrances, sesquiterpenes, α-bulnesene, natural products, total synthesis, ketyl radical anions, butenylation, reductive rearrangement

## Abstract

As a result of its unique fragrance and wider role in traditional medicine, agarwood produced in *Aquilaria* spp. and certain other trees has been harvested to near extinction as a natural phenomenon. Artificially induced agarwood production in *Aquilaria* plantations has sated some of the demand although the product quality is variable. Synthetic chemistry may have a role to play in providing sustainable routes to many of the fragrant components identified in agarwood and its smoke when burnt as incense. In this work, we report efforts towards a total synthesis of the guaiane sesquiterpene α-bulnesene, which is found, along with its more fragrant oxidised derivatives, in agarwood. Following the ring-expansion of (*R*)-carvone using reported procedures, α-butenylation gave a substrate for samarium diiodide mediated reductive cyclisation, the two butenyl epimers of the substrate each leading to a single bicyclic alcohol (**24** and **25**). Overall homoconjugate hydride reduction of one of these alcohols was achieved by Lewis acid-mediated ionisation and then hydride transfer from triethylsilane to complete an overall seven-step synthesis of 5-*epi*-α-bulnesene. This new synthesis paves the way for short routes to both α-bulnesene enantiomers and a study of their aerial and enzymatic oxidation products.

## 1. Introduction

A variety of tree species from, notably, the *Aquilaria* and *Gyrinops* genera (Thymelaeaceae) react to stress by the production of an oleoresin, known inter alia as aloes or agar, that darkens the tree’s heartwood characteristically and progressively with time (years to decades) [1]. This dense, resinous heartwood—agarwood (oud, gahara, jinkoh and others)—has been revered for its unique fragrance for at least 3400 years. Burnt as incense or steam-distilled to form the essential oil, naturally occurring agarwood has become increasingly rare, exacerbated by a growing demand for agarwood objects [2]; the highest grades can sell to aficionados for USD 1000s per gram creating a vicious circle of spiralling demand and cost [3]. Some consequences of this include indiscriminate felling of potential agarwood-bearing trees, to the extent that South-East Asian *Aquilaria* and *Gyrinops* species are now protected under the Convention on International Trade in Endangered Species of Wild Fauna and Flora [4]; a black market of poached, smuggled, adulterated, or fake agarwood [5,6]; agarwood investment schemes (and scams) [7]; and the development of artificial methods of inducing agarwood production in *Aquilaria* plantations by physical, chemical, and biological (fungal inoculation) means [8,9,10,11,12].

The cultural importance, desirability, and high commercial value of agarwood stimulated extensive and ongoing research directed towards understanding the physiological and biochemical processes leading to its production [13] and identifying the chemical components chiefly responsible for the fragrance [14,15,16,17]. As expected, the number and distribution of secondary metabolites identified within different samples of agarwood—including the smoke from smouldering samples, the vapours of heated samples, and the essential oil—vary significantly but, in general, somewhere between 200–400 compounds are present within which sesquiterpenoids and 2-(2-phenylethyl)chromanones dominate [14]. Descriptions of the “characteristic” agarwood fragrance differ, in part due to the variety of sources but also due to inherent subjectivity and social context; typical descriptions include “rich, sweet woody, wet wood-like” [18], “warm, unique balsamic notes with sandalwood-ambergris tonalities” [17], “gorgeous, mysterious and elegant Oriental odor” [19]. The principal contributors to these properties are the relatively volatile sesquiterpenoids, of which 182 are listed in a recent review [14], that span a range of structural types including, especially, eudesmanes, eremophilanes, and guaianes.

**Figure 1:**
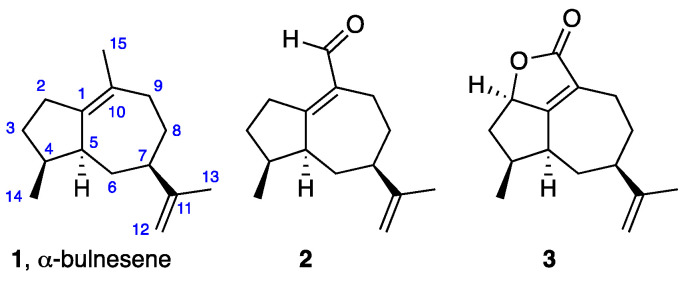
The guaiane sesquiterpene α-bulnesene, showing the accepted numbering system, and two representative fragrant oxidised derivatives found in agarwood.

Our interest in this area stemmed from two observations. First, the oxidation products of α-bulnesene **1** (Figure 1), present in agarwood essential oil and extracts, contribute to the complex fragrance properties; aldehyde **2** and lactone **3** are described in Naef’s influential review [17] as “pleasant, β-damascenone-like, woody, touch of camphor” and “powerful, long lasting, woody, sweet note”, respectively. Zviely and Boix-Camps are more effusive: “a beautiful note of β-damascenone; its woodiness […], almost having soul and spirit” and “its smell is very strong […], extremely long lasting […], clean, radiant, diffusive, bright, woody ambery”, respectively [20]. Second, in our collaborative research on employing engineered P450_BM3_ enzymes in synthesis, we have noted the kinetic resolution of racemic terpenoid substrates [21,22]. Putting these together led us to consider: (a) would the enantiomers of α-bulnesene be discriminated by the Oxford P450_BM3_ library, and (b) would the enantiomers of sesquiterpenoids **2** and **3** have different odours? To answer these questions, a source of both enantiomers of α-bulnesene was sought. 

Τhe naturally occurring enantiomer of α-bulnesene is conveniently available by extraction from patchouli oil [23,24] and has been synthesised [25,26,27,28]. Of the total syntheses, only Heathcock’s route [27] from (±)-Wieland–Miescher ketone could, in principle, be modified to access the separate α-bulnesene enantiomers but, at around 18 steps overall, we aimed to find a shorter route that could be applied equally to both enantiomers. In this report we summarise our study of a free radical cyclisation route to the 7,5-fused ring system leading to 5-*epi*-α-bulnesene in just seven steps from (*R*)-carvone.

## 2. Results and Discussion

**Figure 2:**
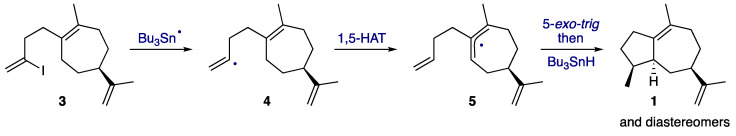
The key step in an initial proposal for the synthesis of α-bulnesene and its diastereomers by homolytic 1,5-hydrogen atom transfer (HAT) and 5-*exo*-*trig* cyclisation.

The synthetic strategy was shaped by our experience in constructing the pyrrolizidine ring system of heliotridane by a 1,5-hydrogen atom transfer/5-*exo-trig* cyclisation [29] which, in turn, had roots in the original work of Parsons [30,31,32] and Curran [33,34]. For α-bulnesene, the initial target was identified as vinyl iodide **3** (Figure 2), a precursor to radical intermediates **4** and then **5**. The regiochemical outcome of the final cyclisation step was not fully predictable, given the lack of close precedent, but C–C bond formation at the methyl-bearing allyl radical terminus was considered to be less favourable on purely steric grounds. The stereochemical outcome was also unclear but the precedent for analogous 5,5- and 6,5-fused ring-forming processes supported the preferred production of the desired *endo*-methyl configuration; see also [35].

**Scheme 1:**
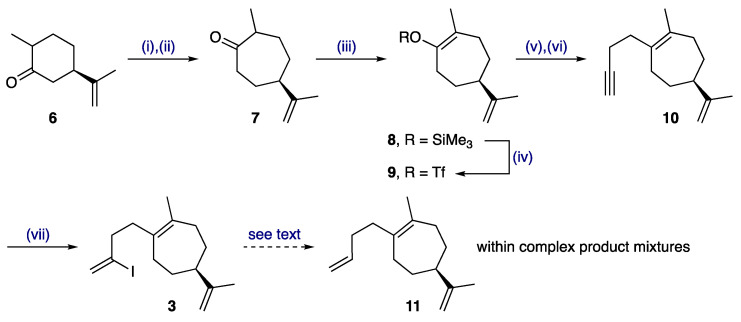
Synthesis of radical precursor **3**. Reagents and conditions: (i) N_2_CHCO_2_Et, BF_3_·OEt_2_, Et_2_O, RT, 2 h; (ii) aq. NaOH, MeOH, reflux, 3 h then aq. HCl (74% from **6**); (iii) MeMgBr, FeCl_3_, Et_2_O, RT, 1 h then **7**, RT, 30 min then Me_3_SiCl, Et_3_N, HMPA, RT, 14 h; (iv) MeLi, THF, RT, 30 min then Comins’ reagent, −78 °C to RT, 2 h (48% from **7**); (v) Me_3_SiC≡C(CH_2_)_2_MgBr, THF, Fe(acac)_3_, NMP, −30 °C, 1 h; (vi) TBAF, THF, RT, 1 h (25%); (vii) DIBAL, Ni(dppp)Cl_2_, THF, 0 °C to RT, 1 h then NIS, 0 °C to RT, 1 h (60%).

A short route was developed (Scheme 1) to vinyl radical precursor **3** that began with ring expansion [36] of dihydro-(*R*)-carvone **6** to afford the analogue **7** as an inconsequential mixture of diastereomers. Regioselective enolisation [37] and silylation, then sulfonylation [38] of the regenerated lithium enolate afforded enol triflate **9** with high (98:2) regioselectivity. The route was completed by employing Fürstner’s variant of the Kumada coupling [39,40] followed by nickel-catalysed hydroalumination and iodination [41]. The low yield in the Kumada coupling step may reflect a slow metal insertion into the C–OTf bond in enol triflate **9** which is co-planar with the adjacent methyl; the analogous reaction of the regioisomeric enol triflate (**S1**, Supplementary Materials) proceeded efficiently (76% yield) despite the volatility of the product.

**Figure 3:**
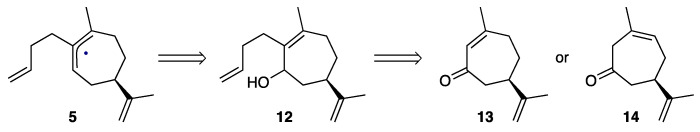
Direct access to the translocated allylic radical **5** from enones **13** and **14**, readily available from (*R*)-carvone.

All attempts to effect the 1,5-HAT/5-*exo-trig* sequence from iodide **3**, or its cycloheptene regiosomer (**S3**, Supplementary Materials), using a variety of conditions based on R_3_SnH and AIBN, returned complex product mixtures whose ^1^H NMR spectra consistently showed resonances corresponding to RCH=CH_2_ [δ_H_ 5.89–5.77 (1H, m), 5.01 (1H, dd, *J* 17.0, 2.0 Hz), 4.94 (1H, dd, *J* 10.5, 2.0 Hz)], indicating simple reduction of the C–I bond (→**11**) as a dominant pathway. A reaction with Bu_3_SnD was considered, in order to identify which of the 1,5-HAT or cyclisation steps was problematic, but this was discarded in light of the complex product profile and poor mass recovery; instead, an unambiguous route to the allylic radical **5** was sought. The ring-expanded derivatives **13** and **14** (Figure 3) of (*R*)-carvone (and their enantiomers from (*S*)-carvone) are available on a multigram scale via efficient three- or four-step sequences. From these, enolate alkylation and carbonyl reduction would provide allylic alcohol **12** from which a variety of radical precursors could potentially be prepared.

**Scheme 2:**
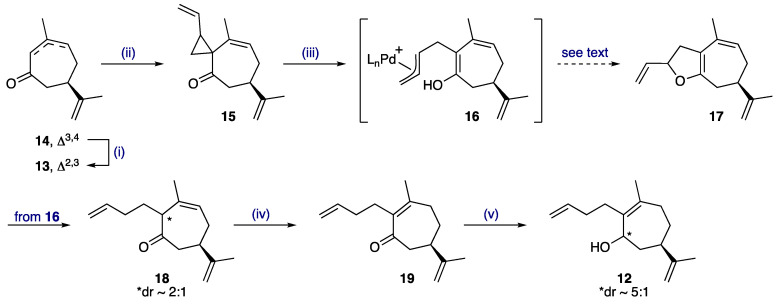
Synthesis of alcohol **12**. Reagents and conditions: (i) DBU, CH_2_Cl_2_, RT, 4 h (95%); (ii) (from **14**) NaH, *t*-BuOK, THF/toluene, RT, 1 h then 1,4-dibromobut-2-ene, −78 °C to RT, 2 h (73%); (iii) Pd_2_(dba)_3_, Bu_3_P, Et_3_N, HCO_2_H, toluene, 105 °C, 2 h (89%); (iv) *t*-BuOK, THF, reflux, 4 h (32% + recovered **18**, 27%); (v) NaBH_4_, CeCl_3_·7H_2_O, MeOH, 0 °C, 1 h (56%).

Cycloheptenone derivative **14** was prepared from (*R*)-carvone as described [42,43] and a sample was isomerised into the conjugated enone isomer **13** under basic conditions (Scheme 2). Direct 3-butenylation of similar ketones has been reported [44] but none of many combinations of base (LHMDS, NaH, *t*-BuONa, KHMDS, *t*-BuOK), solvent (THF, DMF, toluene, *t*-BuOH), additive (DMPU, HMPA), temperature (−78 °C to RT), and electrophile (4-bromobutene, 4-iodobutene) gave acceptable yields of the alkylated product from either cycloheptenone **13** or **14** (Supplementary Materials, Tables S1 and S2). A solution was found in Lautens’ indirect method comprising alkylation with 1,4-dichlorobut-2-ene then Pd(0)-catalysed reductive isomerisation of the so-formed vinyl cyclopropane [45]. Applied to substrate **14**, both stages of this method required much development. The alkylation step was best achieved using a mixed base system for the deprotonation [46] with 1,4-dibromobut-2-ene as an electrophile (Supplementary Materials, Table S3). The reductive rearrangement step was complicated by the presence of the endocyclic C=C bond that presumably stabilises the intermediate π-allyl palladium enol(ate) **16** leading to *O*-alkylation product **17**, sometimes as the major component in the crude product mixture. Eventually, it was found that replacing ammonium formate in the reported procedure with an excess of formic acid and triethylamine eliminated this side reaction and the desired butenylated product **18** was thereby obtained in ~65% yield from ketone **14** (Supplementary Materials, Table S4). Alkene isomerisation (→**19**) and Luche reduction completed the route to alcohol **12**. 

The significant effort expended in achieving a reliable route to the alcohol was not rewarded by success in converting it into a suitable precursor to allylic radical **5**. To summarise many attempts: *O*-thiocarbonyl derivatives showed a strong tendency to undergo [3,3]-shift leading to unreactive *S*-acyl isomers, and intermediates generated en route to the corresponding allylic halides or phenylselenides were generally unstable with respect to elimination leading to volatile cycloheptadienes.

**Figure 4:**
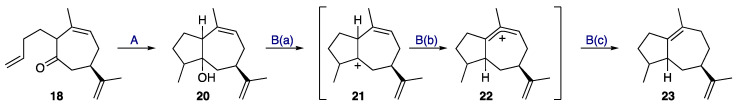
Proposal for a two-step synthesis of α-bulnesene diastereomers from ketone **18** comprising: A—reductive cyclisation; and B—reductive rearrangement.

At this stage in the project, ketones **18** and **19** were identified as candidates for a reductive cyclisation of the derived ketyl radical anion (Step A, Figure 4) for which there is ample precedent [47,48,49,50,51,52]. In the context of the synthesis of africanol and its isomers, the related approaches of Tai [53], Cossy [44], and Piers [54] gave confidence that this could be a reasonably efficient process, with the stereochemical outcome being controllable to a certain extent by the choice of reaction conditions. Following this cyclisation, an ambitious endgame was planned (Step B, Figure 4) which, mechanistically, would involve (a) activation of the bridgehead alcohol to initiate its departure (→**21**), then (b) [1,2]-H shift followed by (c) hydride reduction at the less substituted terminus of the so-formed allylic carbocation **22** leading to the fully substituted alkene **23**. No direct precedent for such a process could be found but the combination of a Brønsted or Lewis acid activator coupled with a trialkylsilane reducing agent [55,56,57,58,59] was envisaged to achieve the transformation. Notable, Carey’s early work [57] with trifluoroacetic acid and trialkylsilanes showed that cations generated from 3°-alcohols are able to undergo stereospecific rearrangement prior to reductive quenching; see also [60].

**Figure 5:**
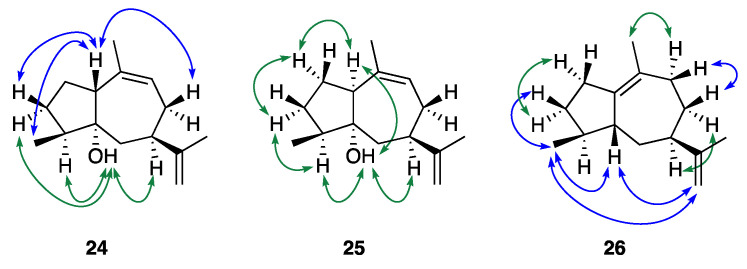
Diagnostic NOE correlations supporting the assigned configuration in reductive cyclisation products **24** and **25**, and the α-bulnesene epimer **26**.

**Scheme 3:**
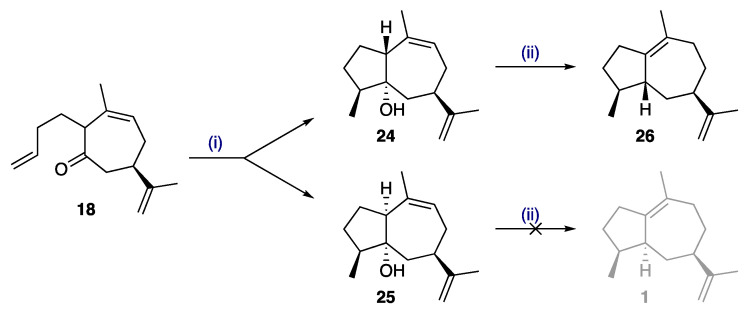
Synthesis of 5-*epi*-α-bulnesene **26**. Reagents and conditions: (i) SmI_2_, *t*-BuOH, HMPA, THF, RT, 30 min (**24**, 35%; **25**, 27%); (ii) Et_3_SiH, BF_3_·OEt_2_, CH_2_Cl_2_, –78 → 0 °C, 1 h (**26**, 54%).

Piers’ experimental conditions [54] for reductive cyclisation were used as the starting point for the analogous reaction of ketone **18** submitted as a mixture of inseparable diastereomers. With minor adjustments to the published procedure (freshly prepared samarium diiodide, less HMPA, a more concentrated solution, shorter reaction time), unwanted side-reactions were minimised and just two major products were isolated (**24** and **25**, Scheme 3), their structures being confirmed by extensive NMR spectroscopic analysis including the key NOE correlations shown (Figure 5; Supplementary Materials, pp. S11–S14). The reaction showed high stereocontrol, with one major product arising from each diastereomer of the starting ketone; notably, both products had the same *trans*-relative disposition of methyl and hydroxyl groups with respect to the fixed isopropenyl substituent. When the separated alcohol diastereomers were treated with boron trifluoride etherate at low temperatures in the presence of an excess of triethylsilane, the outcomes differed dramatically. In the case of *trans*-fused bicyclic alcohol **24**, the reaction proceeded cleanly to deliver a single hydrocarbon **26**, with the merely moderate isolated yield reflecting the high volatility of the product. In contrast, the analogous reaction with *cis*-fused bicyclic alcohol **25** gave no clear products. From these results, it seems reasonable to propose that the departure of the Lewis acid-complexed hydroxyl group in substrate **24** benefits from participation by the antiperiplanar C–H bond leading directly to a relatively long-lived allylic cation; for **25**, no such participation can occur so the localised 3°-carbocation must form fully from which a variety of uncontrolled reactions compete to the exclusion of clean [1,2]-H shift. 

**Figure 6:**
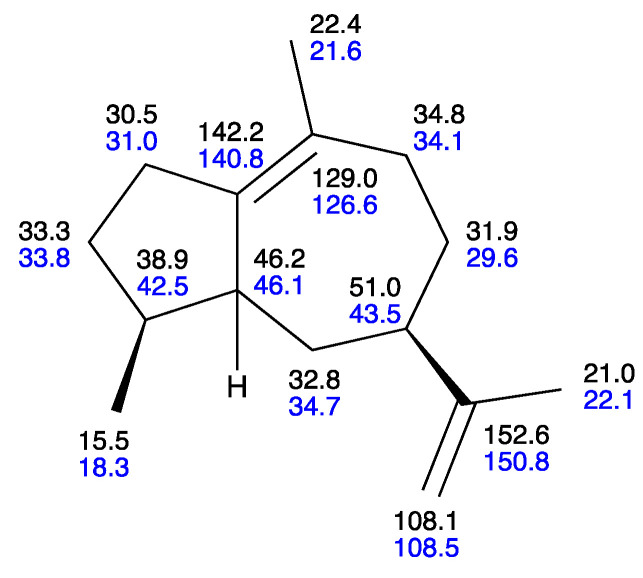
^13^C NMR resonances in CDCl_3_ for α-bulnesene (black), and 5-*epi*-α-bulnesene (blue).

The isolated hydrocarbon **26** is assigned as 5-*epi*-α-bulnesene on the basis of a full NMR spectroscopic analysis; however, very limited NMR data are reported for the epimers of α-bulnesene with which to corroborate the assigned structure. For 5-*epi*-α-bulnesene, ^1^H NMR resonances are available [26] for just =CH_2_ (δ 4.66) and the methyls at C-13 (δ 1.72), C-14 (δ 0.95), and C-15 (δ 1.67); the values obtained for compound **26** are, respectively: δ 4.73/4.70, 1.75, 0.98, and 1.58 with the resonance for CH_3_-15 differing by −0.09 ppm, out of line with the others that differ consistently by +0.03–0.05 ppm, potentially attributable to referencing methods. We considered a number of alternative structures that could reasonably arise from a more complex reductive rearrangement but none of these fit the COSY and HMBC data (Table 1 and Table 2; Supplementary Materials, pp. S15–S17) as well as the assigned structure; indeed, some of these are reported [61] and can be confidently excluded. Furthermore, apart from the C-7 resonance in the ^13^C NMR spectrum, all the ^13^C chemical shifts are within ~3 ppm of the values for authentic α-bulnesene (Figure 6); the aberrant C-7 resonance (43.5 vs. 51.0 for α-bulnesene in CDCl_3_; 43.8 vs. 51.2 for α-bulnesene in C_6_D_6_) is, however, not unreasonable when compared with that reported [62] for 4,5-di-*epi*-α-bulnesene (δ 43.5 in C_6_D_6_).

## 3. Materials and Methods

### 3.1. General Methods

All solvents for anhydrous reactions were obtained dry from Grubbs solvent dispenser units after being passed through an activated alumina column under argon. THF was additionally distilled from sodium/benzophenone ketyl under argon. Commercially available reagents were, in general, used as supplied; amines and dipolar aprotic solvents (HMPA and NMP) were purified by standard methods before use. “Petrol” refers to the fraction of light petroleum ether boiling in the range of 30–40 °C; “ether” refers to diethyl ether. All reactions were carried out in oven-dried glassware and under an inert atmosphere (N_2_ or Ar as specified). Thin layer chromatography (TLC) was carried out using Merck aluminium-backed DC60 F254 0.2 mm precoated plates. Spots were then visualised by the quenching of ultraviolet light fluorescence (λ_max_ 254 nm) and then stained and heated with either anisaldehyde or KMnO_4_ solutions as appropriate. Retention factors (R*_f_*) are reported along with the solvent system used in parentheses. Flash column chromatography was performed using Merck 60 silica gel (particle size 40–63 μm), and the solvent system used is reported in parentheses. Infrared spectra were recorded using a Bruker Tensor 27 FT-IR fitted with a diamond ATR module. Absorption maxima (ν_max_) are reported in wavenumbers (cm^−1^) and are described as strong (s), medium (m), weak (w) or broad (br). Proton (^1^H) and carbon-13 (^13^C) NMR spectra were recorded on Bruker NEO-600 or AVIIIHD-400 spectrometers. Chemical shifts (δ_H_ or δ_C_) are reported in parts per million (ppm) downfield of tetramethylsilane, internally referenced (in MestReNova) to the appropriate solvent peak: CDCl_3_, 7.26/77.16; C_6_D_6_, 7.16/128.06; acetone-*d*_6_, 2.05/206.26. Peak multiplicities are described as singlet (s), doublet (d), triplet (t), quartet (q), quintet (quin), multiplet (m), and broad (br) or a combination thereof. Coupling constants (*J*) are rounded to the nearest 0.5 Hz. Assignments are made on the basis of chemical shifts, integrations, and coupling constants, using COSY, HSQC, HMBC, and NOESY experiments where appropriate. High-resolution mass spectra (HRMS) were recorded by the staff at the Chemistry Research Laboratory (University of Oxford) using a Bruker Daltonics MicroTOF spectrometer; mass-to-charge ratios (*m*/*z*) are reported in Daltons. Melting points were recorded on a Griffin MFB-700-010U melting point apparatus and are uncorrected.

### 3.2. (2RS,5R)-2-Methyl-5-(prop-1-en-2-yl)cycloheptan-1-one (***7***)

BF_3_·OEt_2_ (3.76 mL, 30.0 mmol) was added dropwise to a stirred solution of (2*RS*,5*R*)-dihydrocarvone (3.28 mL, 20.0 mmol) in dry ether (80 mL) under N_2_ at 0 °C. A solution of ethyl diazoacetate (3.16 mL, 30.0 mmol) in dry ether (10 mL) was added over 15 min and the resulting solution was stirred at RT. When the reaction was complete (~2 h, TLC), the mixture was cooled to 0 °C and neutralised with saturated aqueous NaHCO_3_ solution (40 mL). The resulting mixture was extracted with chloroform (3 × 30 mL) then the combined extracts were washed with brine (30 mL), dried (MgSO_4_), filtered, and concentrated. The crude product was purified by flash column chromatography (pentane/ethyl acetate, 25:1) to obtain an intermediate ketoester mixture (4.52 g), which was dissolved in methanol (35 mL) and stirred. A solution of NaOH (1.90 g, 47.4 mmol) in water (40 mL) was added at RT then the mixture was heated at reflux for 3 h, then cooled to RT, diluted with hydrochloric acid (30 mL, 1.0 M), and extracted with ether (3 × 20 mL). The combined extracts were washed successively with water (20 mL) and brine (20 mL), then dried (MgSO_4_), filtered, and concentrated. The residue was purified by chromatography (pentane/ethyl acetate, 20:1) to give the title compound **7** (2.46 g, 74%) as a colourless oil and a 3:1 ratio of unassigned 2-methyl diastereomers. R*_f_*0.40 (pentane/ethyl acetate, 20:1); ν_max_/cm^−1^ 2926 m, 2854 m, 1702 s, 1456 m, 887 m; δ_H_ (400 MHz, CDCl_3_) 4.71–4.63 (2H, m), 2.67–2.41 (3H, m), 2.03–1.94 (1H, m), 1.94–1.78 (3H, m), 1.75–1.55 (4H, m), 1.50–1.36 (2H, m), 1.09 (0.75H, d, *J* = 7.0 Hz), 1.06 (2.25H, d, *J* = 7.0 Hz); δ_C_ (101 MHz, CDCl_3_) (major/minor) 216.5/216.0, 150.7/149.9, 109.0/109.6, 49.3/48.6, 47.0/45.8, 41.6/41.2, 35.0/30.3, 33.3/30.2, 29.9 (both), 20.6/20.9, 17.9/16.1; HRMS (ESI+) *m*/*z* [M + H]^+^ calcd for C_11_H_19_O, 167.1430; found, 167.1432.

### 3.3. (5R)-2-Methyl-5-(prop-1-en-2-yl)cyclohept-1-en-1-yl trifluoromethanesulfonate (***9***)

Methylmagnesium bromide (7.5 mL, 3.0 M in ether, 22.5 mmol) was added dropwise to a stirred suspension of FeCl_3_ (1.20 g, 7.40 mmol) in dry ether (40 mL) under Ar at 0 °C then the mixture was stirred at RT for 1 h. A solution of ketone **7** (830 mg, 5.00 mmol) in dry ether (5 mL) was added dropwise, the resulting suspension was stirred for 30 min and then trimethylsilyl chloride (1.90 mL, 15.0 mmol), triethylamine (2.10 mL, 15.1 mmol), and HMPA (0.870 mL, 5.00 mmol) were added sequentially. The mixture was stirred for 14 h at RT then diluted with ether (40 mL), washed with cold saturated aqueous NaHCO_3_ solution (2 × 10 mL), dried (MgSO_4_), filtered, and concentrated. The crude silyl enol ether was dissolved in dry THF (40 mL) and cooled to 0 °C under Ar. Methyllithium (4.4 mL, 1.6 M in ether, 7.0 mmol) was added dropwise then the mixture was allowed to warm to RT and stirred for 30 min. The solution was re-cooled to −78 °C, a solution of Comins’ reagent (3.1 g, 7.9 mmol) in THF (10 mL) was added dropwise, and then the mixture was allowed to warm slowly to RT and stirred for a further 2 h. The solvent was removed under vacuum and the residue was purified by chromatography (pentane) to give enol triflate **9** as a yellow oil (721 mg, 48%). R*_f_* 0.38 (pentane); [α]_D_^25^ +3.8 (*c* 0.62, CHCl_3_); ν_max_/cm^−1^ 2937 m, 1413 m, 1245 m, 1207 s, 1143 s; δ_H_ (400 MHz, CDCl_3_) 4.70 (1H, quin, *J* = 1.5 Hz), 4.68 (1H, br s), 2.58 (1H, br app t, *J* = 14.0 Hz), 2.46 (1H, dd, *J* = 16.0, 6.5 Hz), 2.26–2.15 (2H, m), 2.11 (1H, tt, *J* = 10.5, 4.0 Hz), 1.86–1.75 (2H, m) overlaying 1.81 (3H, d, *J* = 2.0 Hz), 1.71 (3H, s), 1.60–1.51 (1H, m), 1.46–1.39 (1H, m); δ_C_ (151 MHz, CDCl_3_) 150.2, 146.2, 131.6, 118.5 (q, *J* = 319.5 Hz), 109.3, 49.2, 32.0, 31.5, 30.1, 29.7, 21.1, 19.5; HRMS (APCI–) *m*/*z* [M–H^+^]^−^ calcd for C_12_H_16_O_3_F_3_S, 297.0778; found, 297.0765.

### 3.4. (5R)-1-(But-3-yn-1-yl)-2-methyl-5-(prop-1-en-2-yl)cyclohept-1-ene (***10***)

A solution of [4-(trimethylsilyl)but-3-yn-1-yl]magnesium bromide in THF was prepared by adding (4-bromobut-1-yn-1-yl)trimethylsilane (156 mg, 0.760 mmol) into a stirred suspension of Mg (184 mg, 7.57 mmol) in dry THF (10 mL). After formation of the grey Grignard solution was complete, it was added into a −30 °C solution of enol triflate **9** (113 mg, 0.379 mmol), Fe(acac)_3_ (7.0 mg, 0.02 mmol), and NMP (0.30 mL, 3.1 mmol) in dry THF (10 mL) under N_2_, leading to an immediate colour change from orange to black. The mixture was stirred for 1 h at −30 °C and was then quenched with saturated aqueous NH_4_Cl solution (10 mL). The aqueous phase was extracted with ether (3 × 10 mL) then the combined organic extracts were dried (Na_2_SO_4_), filtered, and concentrated. The residue was dissolved in dry THF (5 mL) then TBAF (0.19 mL, 1.0 M in THF, 0.19 mmol) was added and the mixture stirred under N_2_ for 1 h at RT. Saturated aqueous NH_4_Cl solution (10 mL) was added and the aqueous layer was extracted with ether (3 × 10 mL). The combined organic extracts were dried (MgSO_4_), filtered, and concentrated. The residue was purified by column chromatography (pentane) to give the title compound **10** as a colourless oil (19 mg, 25%). This volatile compound was used directly in the next step. R*_f_* 0.42 (pentane); δ_H_ (400 MHz, CDCl_3_) 4.64–4.62 (2H, m), 2.37–2.16 (6H, m), 2.11 (1H, tt, *J* = 11.5, 3.5 Hz), 2.08–1.94 (2H, m), 1.92 (1H, t, *J* = 2.5 Hz), 1.75–1.65 (2H, m) overlaying 1.72 (3H, d, *J* = 1.5 Hz) and 1.68 (3H, t, *J* = 1.0 Hz), 1.25–1.12 (2H, m); δ_C_ (101 MHz, CDCl_3_) 151.9, 134.4, 134.0, 108.4, 84.8, 68.2, 51.7, 34.9, 34.4, 32.8, 32.0, 31.2, 21.1, 20.9, 17.7.

### 3.5. (5R)-1-(3-Iodobut-3-en-1-yl)-2-methyl-5-(prop-1-en-2-yl)cyclohept-1-ene (***3***)

DIBAL (0.72 mL, 1.2 M in toluene, 0.86 mmol) was added dropwise to a solution of Ni(dppp)Cl_2_ (47 mg, 0.087 mmol) in dry THF (10 mL) under Ar. The mixture was cooled to 0 °C and alkyne **10** (88.0 mg, 0.435 mmol) was added via syringe into the black solution; the mixture was warmed to RT and stirred for 1 h then cooled to 0 °C. A solution of *N*-iodosuccinimide (292 mg, 1.30 mmol) in dry THF (2 mL) was added dropwise, then the solution was warmed to RT and stirring continued for a further 1 h. The solution was quenched with saturated aqueous Rochelle’s salt solution (10 mL), the aqueous phase was extracted with ether (3 × 20 mL), and the combined organic extracts were dried (MgSO_4_), filtered, and concentrated. The residue was purified by column chromatography (pentane/triethylamine, 100:1) to give vinyl iodide **3** as a colourless oil (86.5 mg, 60%). R*_f_* 0.25 (pentane); [α]_D_^25^ + 8.3 (*c* 0.29, CHCl_3_); ν_max_/cm^–1^ 2981 s, 1382 m, 1252 m, 1151 m, 955 m; δ_H_ (400 MHz, CDCl_3_) 6.00 (1H, q, *J* = 1.5 Hz), 5.68 (1H, d, *J* = 1.5 Hz), 4.64 (2H, br s), 2.50–2.40 (2H, m), 2.33–2.20 (4H, m), 2.15–2.08 (1H, tt, *J* = 11.5, 3.5 Hz), 2.08–1.95 (2H, m), 1.75–1.65 (2H, m) overlaying 1.72 (3H, d, *J* = 1.5 Hz) and 1.68 (3H, t, *J* = 1.0 Hz), 1.21–1.08 (2H, m); δ_C_ (101 MHz, CDCl_3_) 151.8, 134.0, 133.8, 125.3, 112.3, 108.4, 51.7, 44.5, 35.5, 34.9, 33.1, 32.1, 31.3, 21.2, 21.0; HRMS (ESI+) *m*/*z* [M + H]^+^ calcd for C_15_H_24_I, 331.0917; found, 331.0916.

### 3.6. (6R)-3-Methyl-6-(prop-1-en-2-yl)cyclohept-2-en-1-one (***13***)

DBU (0.40 mL, 2.7 mmol) was added to a stirred solution of cycloheptenone derivative **14** (420 mg, 2.56 mmol) in dichloromethane (13 mL) at RT under N_2_. The mixture was stirred for 3 h and then quenched with the addition of hydrochloric acid (5 mL, 1.0 M). The layers were separated, and the aqueous layer was extracted with dichloromethane (2 × 10 mL) then the combined organic portions were washed with brine (10 mL), dried (MgSO_4_), filtered, and concentrated to yield the title compound **13** (400 mg, 95%), a colourless oil, that was sufficiently pure to use in further steps. A sample was purified for analytical purposes by column chromatography (pentane/ether, 9:1). The analytical data are as reported in [63].

### 3.7. (6R)-9-Methyl-6-(prop-1-en-2-yl)-1-vinylspiro[2.6]non-8-en-4-one (***15***)

Potassium *tert*-butoxide (4.0 mL, 1.0 M in THF, 4.0 mmol) was added to a RT suspension of NaH (267 mg, 60% in mineral oil, 6.68 mmol) in dry toluene (80 mL) under Ar and the mixture was stirred for 1 h. A solution of ketone **14** (550 mg, 3.35 mmol) in dry toluene (10 mL) was added via syringe, stirring was continued for 1 h then the enolate solution was cooled to −78 °C and a solution of (*E*)-1,4-dibromo-2-butene (2.86 g, 13.4 mmol) in dry toluene (20 mL) was added dropwise over 10 min. The mixture was warmed slowly to RT and stirred for 2 h then quenched with saturated aqueous NH_4_Cl solution (20 mL) and extracted with ether (3 × 20 mL). The combined extracts were washed with brine (20 mL), dried (MgSO_4_), filtered, and concentrated to give a residue that was purified by chromatography (pentane/ethyl acetate, 20:1), affording the product **15** as a yellow oil and as a pair of unassigned diastereomers (527 mg, 73%, dr = 1:1). R*_f_* 0.54 (pentane/ethyl acetate, 20:1); ν_max_/cm^–1^ 2966 m, 2917 m, 1686 s, 1645 m, 1439 m; δ_H_ (400 MHz, CDCl_3_) 5.85–5.71 (2H, m), 5.21 (0.5H, dd, *J* = 17.0, 2.0 Hz), 5.20 (0.5H, dd, *J* = 17.0, 2.0 Hz), 5.04 (0.5H, dd, *J* = 10.5, 2.0 Hz), 5.02 (0.5H, dd, *J* = 10.5, 2.0 Hz), 4.76–4.73 (1H, m), 4.73–4.68 (1H, m), 2.84–2.69 (1.5H, m), 2.64 (1H, d, *J* = 7.0 Hz), 2.51–2.43 (1H, m), 2.30–2.24 (1H, m), 2.21–2.11 (1H, m), 1.98 (0.5H, app. q, *J* = 8.5 Hz), 1.90 (0.5H, dd, *J* = 7.5, 4.5 Hz), 1.76 (0.5H, dd, *J* = 7.5, 5.0 Hz), 1.72 (1.5H, br s), 1.70 (1.5H, br s), 1.69 (1.5H, br s), 1.67 (1.5H, q, *J* = 1.0 Hz), 1.44 (0.5H, dd, *J* = 8.5, 5.0 Hz), 1.31 (0.5H, dd, *J* = 9.0, 4.5 Hz); δ_C_ (101 MHz, CDCl_3_) 209.3/208.7, 148.4/148.2, 137.5/136.0, 135.8/135.4, 126.4/125.9, 116.5 (both), 110.2/109.9, 46.5/45.9, 46.2/42.7, 42.9/42.2, 36.8/36.0, 31.2/29.4, 21.9/21.1, 21.2/21.0, 20.7 (two peaks); HRMS (ESI+) *m*/*z* [M + H]^+^ calcd for C_15_H_21_O, 217.1587; found, 217.1586.

### 3.8. (2RS,6R)-2-(But-3-en-1-yl)-3-methyl-6-(prop-1-en-2-yl)cyclohept-3-en-1-one (***18***)

Triethylamine (1.36 mL, 9.76 mmol) and then formic acid (1.29 mL, 34.2 mmol) were added to a RT solution of Pd_2_(dba)_3_ (45 mg, 0.049 mmol) and tributylphosphine (98 μL, 0.39 mmol) in dry toluene (35 mL) under Ar. The resulting suspension was stirred for 5 min and then heated to 105 °C. A solution of ketone **15** (526 mg, 2.43 mmol) in dry toluene (5 mL) was added dropwise into the heated mixture over 5 min and stirring was continued at 105 °C for 2 h. The mixture was cooled to RT and water (20 mL) was added. The resulting mixture was extracted with ether (3 × 20 mL), and the combined extracts were washed with brine (20 mL), dried (MgSO_4_), filtered, and concentrated. The residue was purified by chromatography (pentane/ethyl acetate, 30:1) to give the title compound **18**, a pair of diastereomers (468 mg, 89%, dr = 2:1), as a yellow oil. R*_f_* 0.31 (pentane/ethyl acetate, 30:1); ν_max_/cm^–1^ 2935 m, 2857 m, 1707 s, 1643 m, 1447 m; δ_H_ (400 MHz, CDCl_3_) (indicative integrals) 5.85–5.74 (1H, m), 5.69–5.57 (1H, m), 5.06–4.93 (2H, m), 4.80–4.67 (2H, m), 3.26 (0.67H, dd, *J* = 8.5, 4.5 Hz), 3.11 (0.33H, dd, *J* = 8.5, 5.0 Hz), 2.72–2.62 (2.67H, m), 2.55–2.48 (0.67H, m), 2.37–2.25 (0.67H, m), 2.26–2.13 (1.67H, m), 2.09–2.00 (1.67H, m), 2.00–1.92 (0.67H, m), 1.77 (1H, br s), 1.75 (2H, br s), 1.73–1.72 (3H, m) overlaying 1.82–1.60 (1H, m); δ_C_ (101 MHz, CDCl_3_) (major/minor) 209.5/210.1, 148.2/148.4, 138.4/138.2, 135.9/135.5, 125.3/124.9, 115.2/115.3, 110.0/110.2, 56.0/57.0, 48.9/46.9, 47.4/45.2, 31.9/31.8, 30.5/31.7, 26.9/27.7, 22.2/24.2, 20.6/20.8; HRMS (ESI+) *m*/*z* [M + H]^+^ calcd for C_15_H_23_O, 219.1743; found, 219.1745.

### 3.9. (6R)-2-(But-3-en-1-yl)-3-methyl-6-(prop-1-en-2-yl)cyclohept-2-en-1-one (***19***)

A solution of potassium *tert*-butoxide (0.030 mL, 1.0 M in THF, 0.030 mmol) was added to a RT solution of ketone **18** (22 mg, 0.10 mmol) in dry toluene (2 mL) under N_2_. The mixture was heated to 90 °C and stirred for 4 h. The resulting orange solution was cooled to RT and quenched with saturated aqueous NH_4_Cl solution (5 mL) then extracted with ether (3 × 5 mL). The combined organic phases were dried (Na_2_SO_4_), filtered, and concentrated. The residue was purified by chromatography (pentane/ethyl acetate, 30:1) to give the ketone **19** (7.0 mg, 32%) as a colourless oil and recovered ketone **18** (6.0 mg, 27%). R*_f_* 0.22 (pentane/ethyl acetate, 10:1); [α]_D_^25^ +59.8 (*c* 0.22, CHCl_3_); ν_max_/cm^–1^ 2980 s, 1660 s, 1447 m, 1376 m, 908s; δ_H_ (400 MHz, CDCl_3_) 5.76 (1H, ddt, *J* = 17.0, 10.0, 7.0 Hz), 4.97 (1H, dq, *J* = 17.0, 1.5 Hz), 4.91 (1H, br d, *J* = 10 Hz)), 4.73 (2H, br s), 2.61–2.56 (2H, m), 2.54–2.45 (2H, m), 2.44–2.31 (2H, m), 2.23 (1H, ddd, *J* = 16.5, 6.0, 3.0 Hz), 2.10–2.02 (2H, m), 1.92 (3H, s), 1.88–1.74 (1H, m), 1.72 (3H, br s), 1.66 (1H, dddd, *J* = 14.0, 7.5, 6.0, 4.0 Hz); δ_C_ (101 MHz, CDCl_3_) 205.4, 150.0, 148.0, 138.5, 137.9, 114.9, 109.9, 46.8, 40.2, 33.7, 33.5, 29.8, 28.4, 23.0, 21.1; HRMS (ESI+) *m*/*z* [M + H]^+^ calcd for C_15_H_23_O, 219.1743; found, 219.1743.

### 3.10. (1RS,6R)-2-(But-3-en-1-yl)-3-methyl-6-(prop-1-en-2-yl)cyclohept-2-en-1-ol (***12***)

NaBH_4_ (11 mg, 0.29 mmol) was added to a stirred solution of CeCl_3_·7H_2_O (109 mg, 0.293 mmol) and ketone **19** (58.0 mg, 0.268 mmol) in methanol (25 mL) at 0 °C under N_2_. Stirring was continued at 0 °C for 1 h by which time the reaction was complete (TLC). Water (10 mL) was added, and the mixture was extracted with ether (4 × 20 mL); the combined organic extracts were washed with brine (20 mL), dried (MgSO_4_), filtered, and concentrated. The residue was purified by chromatography (pentane/ethyl acetate, 10:1) to give the title compound **12** (33 mg, 56%) as a colourless solid, and as a pair of unassigned diastereomers (dr~5:1). R*_f_* 0.35 (pentane/ethyl acetate, 10:1); m.p. 41 °C; ν_max_/cm^–1^ 3661w, 2981 s, 2890 m, 1461 m, 1153 m; δ_H_ (600 MHz, C_6_D_6_) (major diastereomer) 5.94 (1H, ddt, *J =* 17.0, 10.0, 6.5 Hz), 5.12 (1H, dq, *J* = 17.0, 1.5 Hz), 5.01 (1H, br d, *J* = 10.0 Hz), 4.74 (2H, br s), 4.28 (1H, d, *J* = 10.0 Hz), 2.45–2.35 (2H, m), 2.34–2.23 (2H, m), 2.11 (1H, tt, *J* = 12.0, 4.0 Hz), 2.07–2.01 (1H, m), 1.82 (1H, ddd, *J* = 14.5, 7.0, 1.5 Hz), 1.64 (3H, d, *J* = 1.5 Hz), 1.60 (3H, t, *J* = 1.0 Hz), 1.58–1.47 (3H, m), 1.04 (1H, dtd, *J* = 13.5, 12.0, 1.5 Hz); δ_C_ (151 MHz, C_6_D_6_) (major diastereomer) 150.7, 139.6, 138.8, 130.5, 114.5, 109.3, 72.0, 49.0, 43.1, 34.7, 33.9, 30.5, 28.0, 21.3, 20.6; HRMS (ESI+) *m*/*z* [M + H–H_2_O]^+^ calcd for C_15_H_23_, 203.1794; found, 203.1794.

### 3.11. (3S,3aR,5R,8aR)-3,8-Dimethyl-5-(prop-1-en-2-yl)-2,3,4,5,6,8a-hexahydroazulen-3a(1H)-ol (***24***); (3S,3aR,5R,8aS)-3,8-dimethyl-5-(prop-1-en-2-yl)-2,3,4,5,6,8a-hexahydroazulen-3a(1H)-ol (***25***)

1,2-Diiodoethane (387 mg, 1.37 mmol) was added to a stirred suspension of Sm powder (360 mg, 2.39 mmol) in dry THF (14 mL) under Ar. The resulting solution was stirred for 2 h to yield a deep blue SmI_2_ solution. Dry HMPA (1.0 mL, 5.7 mmol) was then added to generate a deep purple solution. A solution of ketone **18** (120 mg, 0.555 mmol) and dry *tert*-butanol (1.05 ml, 1.10 mmol) in dry THF (3 mL) was transferred into the HMPA/SmI_2_ solution dropwise by syringe over 5 min and the mixture was stirred for 30 min. Saturated aqueous NaHCO_3_ solution (10 mL) was added, the mixture was extracted with ether (3 × 10 mL), and the combined extracts were washed with brine (10 mL), then dried (MgSO_4_), filtered, and concentrated. The residue was purified by chromatography on basic alumina (pentane/ethyl acetate, 30:1) to give the *trans*-fused bicyclic alcohol **24** (43 mg, 35%) as a yellow oil. R*_f_* 0.56 (pentane/ethyl acetate, 10:1); [α]_D_^25^ +30.7 (*c* 0.25, CH_2_Cl_2_); IR (film) ν_max_/cm^–1^ 3567w, 2934 s, 2854 m, 1644 m, 1446 s; δ_H_ (600 MHz, acetone-*d*_6_) 5.74–5.70 (1H, m, H9), 4.68–4.67 (1H, m, H12), 4.64–4.62 (1H, m, H12′), 2.77 (1H, br t, *J* = 9.5 Hz, H1), 2.60 (1H, s, OH), 2.27 (1H, br t, *J* = 11.0 Hz, H7), 2.21–2.12 (1H, m, H8), 2.09–1.95 (3H, m, H3, H4, H8′), 1.92–1.84 (2H, m, H2, H6), 1.78–1.72 (1H, m, H2′), 1.71–1.70 (6H, m, H13, H15), 1.64 (1H, dd, *J* = 13.5, 12.0 Hz, H6′), 1.12 (1H, ddt, *J* = 12.0, 9.5, 7.0 Hz, H3′), 0.93 (3H, d, *J* = 7.0 Hz, H14); δ_C_ (151 MHz, acetone-*d*_6_) 153.0 (C11), 139.3 (C10), 126.3 (C9), 108.9 (C12), 79.3 (C5), 51.0 (C1), 47.6 (C4), 46.6 (C6), 39.8 (C7), 34.2 (C8), 33.8 (C3), 27.2 (C2), 23.7 (C15), 20.8 (C13), 17.6 (C14); HRMS (ESI+) *m*/*z* [M + H–H_2_O]^+^ calcd for C_15_H_23_, 203.1794; found, 203.1796. The *cis*-fused bicyclic alcohol **25** was also obtained as a yellow oil (33 mg, 27%). R*_f_* 0.30 (pentane/ethyl acetate, 10:1); [α]_D_^25^ +46.5 (*c* 0.11, CH_2_Cl_2_); ν_max_/cm^–1^ 3447w, 2923s, 1457m; δ_H_ (600 MHz, acetone-*d*_6_) 5.51 (1H, tq, *J* = 6.0, 1.5 Hz, H9), 4.68–4.65 (1H, m, H12), 4.61–4.60 (1H, m, H12′), 3.27 (1H, br s, OH), 2.55–2.49 (1H, m, H7), 2.43 (1H, dd, *J* = 12.5, 8.5 Hz, H1), 2.09–2.05 (2H, m, H8), 1.98–1.85 (3H, m, H2, H3, H4), 1.71 (3H, br s, H13) and 1.70 (3H, br s, H15) overlays 1.72–1.68 (1H, m, H6), 1.59 (1H, dd, *J* = 13.5, 11.5 Hz, H6′), 1.59–1.52 (1H, m, H2′), 1.29–1.21 (1H, m, H3′), 0.87 (3H, d, *J* = 6.5 Hz, H14); δ_C_ (151 MHz, acetone-*d*_6_) 153.5 (C11), 138.6 (C10), 123.8 (C9), 108.6 (C12), 78.8 (C5), 57.6 (C1), 47.4 (C4), 39.6 (C7), 38.8 (C6), 35.1 (C8), 29.0 (C3), 28.1 (C15), 26.6 (C2), 21.0 (C13), 14.1 (C14); HRMS (ESI+) *m*/*z* [M + H–H_2_O]^+^ calcd for C_15_H_23_, 203.1794; found, 203.1795.

### 3.12. (3S,3aR,5R)-3,8-Dimethyl-5-(prop-1-en-2-yl)-1,2,3,3a,4,5,6,7-octahydroazulene [5-epi-α-bulnesene] (***26***)

A solution of BF_3_·OEt_2_ (0.0152 mL, 0.120 mmol) in dry dichloromethane (1.52 mL) was added dropwise over 5 min to a −78 °C solution of alcohol **24** (39.0 mg, 0.177 mmol) and triethylsilane (0.168 mL, 1.05 mmol) in dry dichloromethane (2 mL) under N_2_. The resulting mixture was allowed to warm slowly to 0 °C and was then stirred for 1 h. Saturated aqueous NaHCO_3_ solution (10 mL) was added, and the mixture was extracted with ether (3 × 10 mL). The combined organic extracts were dried (MgSO_4_), filtered, and concentrated carefully under vacuum. The residue was purified by chromatography (pentane) to give the title compound **26** (19.5 mg, 54%) as a volatile, colourless oil. R*_f_* 0.63 (pentane); [α]_D_^25^ + 40.8 (*c* 0.95, CH_2_Cl_2_); ν_max_/cm^–1^ 2923 s, 2866 s, 1643 m, 1456 m, 1375 m; δ_H_ (600 MHz, CDCl_3_) 4.73 (1H, br s, H12), 4.70 (1H, br s, H12′), 2.35–2.30 (1H, m, H7), 2.25 (1H, dd, *J* = 16.5, 8.5 Hz, H2), 2.21–2.10 (3H, m, H2′, H9), 2.05 (1H, tq, *J* = 10.0, 2.5 Hz, H5), 1.89–1.82 (1H, m, H8), 1.82–1.77 (2H, m, H3, H6), 1.75 (3H, br s, H13), 1.72–1.65 (1H, m, H6′), 1.65–1.59 (1H, m, H8′), 1.58 (3H, br s, H15), 1.41–1.32 (1H, m, H4), 1.13 (1H, qd, *J* = 11.5, 8.5 Hz, H3′), 0.99 (3H, d, *J* = 6.5 Hz, H14); δ_C_ (151 MHz, CDCl_3_) 150.8 (C11), 140.8 (C1), 126.6 (C10), 108.5 (C12), 46.1 (C5), 43.5 (C7), 42.5 (C4), 34.7 (C6), 34.1 (C9), 33.8 (C3), 31.0 (C2), 29.6 (C8), 22.1 (C13), 21.6 (C15), 18.3 (C14); HRMS (EI) *m*/*z* [M]^•+^ calcd for C_15_H_24_, 204.1873; found, 204.1878.

## 4. Conclusions

**Figure 7:**
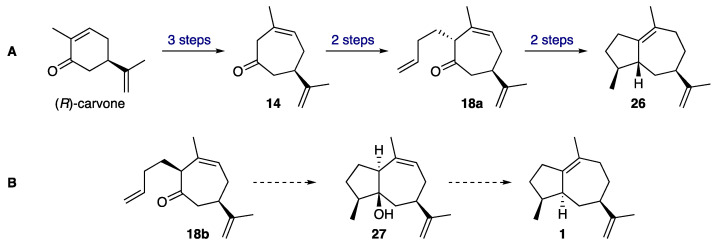
(**A**)—Summary of the synthesis of 5-*epi*-α-bulnesene from (*R*)-carvone. (**B**)—Projected route to α-bulnesene.

This study set out to develop a short synthesis of both enantiomers of α-bulnesene in order to investigate their relative reactivity towards our local library of P450_BM3_ enzymes and the fragrance properties of oxidised derivatives in both enantiomeric series. The initially planned 1,5-HAT/cyclisation strategy was not productive but led to the exploration of a related ketyl radical anion cyclisation. During this project, optimised conditions were developed for overall butenylation of enone **14**; a modified reductive cyclisation protocol led to increased efficiency and stereoselectivity compared with previously reported close precedents, and a new reductive rearrangement step completed the synthesis not of α-bulnesene itself but of the 5-*epi*-isomer. The overall route (Figure 7A) from carvone is short (seven steps) and sufficiently efficient (~15%) for the project’s purposes. Current work is exploring methods [64,65,66] to produce the “missing stereoisomer” **27** projected to lead to the natural product (Figure 7B) and, from (*S*)-carvone, its enantiomer.

## Figures and Tables

**Table 1:** Observed ^1^H,^1^H-COSY correlations for compound **26**.

Proton	Correlated with
H_2_-2	H_2_-3, H_3_-15
H_2_-3	H_2_-2, H-4
H-4	H_2_-3, H-5, H_3_-14
H-5	H-4, H_2_-6, H_3_-15
H_2_-6	H-5, H-7
H-7	H_2_-6, H_2_-8, H_3_-15
H_2_-8	H-7, H_2_-9 ^1^
H_2_-9	overlapped ^1^
H_2_-12	H-7, H_3_-13
H_3_-13	H_2_-12
H_3_-14	H-4
H_3_-15	H_2_-2, H-5

^1^ Ambiguous correlation between overlapping multiplets.

**Table 2:** Observed ^1^H,^1^H-COSY and ^1^H,^13^C-HMBC correlations for compound **26** ^1^.

Carbon	Expected ^2^*J*_C–H_	Expected ^3^*J*_C–H_
C-1	H_2_-2, H-5	*H_2_-3*, (H-4), ^2^ H_2_-6, *H_2_-9*, H-15
C-2	H_2_-3	(H-4), ^2^ (H-5) ^3^
C-3	H_2_-2, H-4	(H-5), ^3^ H_3_-14
C-4	H_2_-3, H-5, H_3_-14	H_2_-2, H_2_-6
C-5	H-4, H_2_-6	H_2_-2, H_2_-3, H-7, H_3_-14
C-6	(H-5), ^3^ H-7	H-4, H_2_-8
C-7	H_2_-6, H_2_-8	(H-5), ^3^ *H_2_-9*, H_2_-12, H_3_-13
C-8	H-7, *H_2_-9*	H_2_-6
C-9	H_2_-8	H-7, H_3_-15
C-10	*H_2_-9*, H_3_-15	H_2_-2, H-5, H_2_-8
C-11	H-7, H_2_-12, H_3_-13	H_2_-6, H_2_-8
C-12	—	H-7, H_3_-13
C-13	—	H-7, H_2_-12
C-14	H-4	H_2_-3, (H-5) ^3^
C-15	—	*H_2_-9*

^1^ All expected correlations are observed except those in parentheses. Ambiguous correlations with overlapping ^1^H multiplets are italicised (this mainly concerns H_2_-9 that is wholly contained within an unresolved multiplet overlapping with one of H_2_-2). ^2^ C-4 shows expected correlation to H_2_-2. ^3^ C-5 shows expected correlations to H_2_-2, H_2_-3, H_2_-6, H-7, and H_3_-14.

## Data Availability

Not applicable.

## Supplementary Materials

The following supporting information can be downloaded at: https://www.mdpi.com/article/10.3390/molecules28093900/s1. NMR spectra for compounds in Scheme 1, Scheme 2 and Scheme 3, reference NMR data for α-bulnesene **1**, procedures and characterisation data for **S1**–**S3**, optimisation tables for the formation of butenyl ketone **18** (PDF).

## Sample Availability

Samples of the compounds are not available from the authors.
