# Supplementary material for: An Enantiospecific Synthesis of 5-epi-α-Bulnesene
Source: Molecules. 2023 May 5;28(9):3900. doi: 10.3390/molecules28093900 (PMC10180261; doi:10.3390/molecules28093900)
Supplement: Supplementary file 1 [file molecules-28-03900-s001.zip › molecules-2381776-supplementary.pdf]

An Enantiospecific Synthesis of 5-*epi*-α-Bulnesene

Jiarui Zong and Jeremy Robertson*

* Correspondence: jeremy.robertson@chem.ox.ac.uk

NMR spectra **Page**

(2RS,5R)-2-Methyl-5-(prop-1-en-2-yl)cycloheptan-1-one (7)
^{1}H NMR spectrum S3
^{13}C NMR spectrum S3
*(5R)-2-Methyl-5-(prop-1-en-2-yl)cyclohept-1-en-1-yl trifluoromethanesulfonate (**9**)*
^{1}H NMR spectrum S4
^{13}C NMR spectrum S4
*(5R)-1-(But-3-yn-1-yl)-2-methyl-5-(prop-1-en-2-yl)cyclohept-1-ene (**10**)*
^{1}H NMR spectrum S5
^{13}C NMR spectrum S5
*(5R)-1-(3-Iodobut-3-en-1-yl)-2-methyl-5-(prop-1-en-2-yl)cyclohept-1-ene (**3**)*
^{1}H NMR spectrum S6
^{13}C NMR spectrum S6
*(6R)-9-Methyl-6-(prop-1-en-2-yl)-1-vinylspiro[2.6]non-8-en-4-one (**15**)*
^{1}H NMR spectrum S7
^{13}C NMR spectrum S7
*(2RS,6R)-2-(But-3-en-1-yl)-3-methyl-6-(prop-1-en-2-yl)cyclohept-3-en-1-one (**18**)*
^{1}H NMR spectrum S8
^{13}C NMR spectrum S8
*(6R)-2-(But-3-en-1-yl)-3-methyl-6-(prop-1-en-2-yl)cyclohept-2-en-1-one (**19**)*
^{1}H NMR spectrum S9
^{13}C NMR spectrum S9
*(1RS,6R)-2-(But-3-en-1-yl)-3-methyl-6-(prop-1-en-2-yl)cyclohept-2-en-1-ol (**12**)*
^{1}H NMR spectrum S10
^{13}C NMR spectrum S10
*(3S,3aR,5R,8aR)-3,8-Dimethyl-5-(prop-1-en-2-yl)-2,3,4,5,6,8a-hexahydroazulen-3a(1H)-ol (**24**)*
^{1}H NMR spectrum S11
^{13}C NMR spectrum S11
HSQC spectrum S12
NOESY spectrum S12
*(3S,3aR,5R,8aS)-3,8-dimethyl-5-(prop-1-en-2-yl)-2,3,4,5,6,8a-hexahydroazulen-3a(1H)-ol (**25**)*
^{1}H NMR spectrum S13
^{13}C NMR spectrum S13
HSQC spectrum S14
NOESY spectrum S14
*(3S,3aR,5R)-3,8-Dimethyl-5-(prop-1-en-2-yl)-1,2,3,3a,4,5,6,7-octahydroazulene [5-epi-α-bulnesene] (**26**)*
^{1}H NMR spectrum S15
^{13}C NMR spectrum S15
HSQC spectrum S16
HMBC spectrum S16
NOESY spectrum S17
*(3S,3aR,5S)-3,8-Dimethyl-5-(prop-1-en-2-yl)-1,2,3,3a,4,5,6,7-octahydroazulene [α-bulnesene] (**1**)*
^{1}H NMR spectrum S18
^{13}C NMR spectrum S18

Procedures, characterisation data, and NMR spectra for the alkene regioisomers of compounds 9, 10, and 13 [S1, S2, and S3, respectively]

Procedures S19

*(7RS,4R)-7-Methyl-4-(prop-1-en-2-yl)cyclohept-1-en-1-yl trifluoromethanesulfonate (**S1**)*
^{1}H NMR spectrum S21
^{13}C NMR spectrum S21
*(7RS,4R)-1-(But-3-yn-1-yl)-7-methyl-4-(prop-1-en-2-yl)cyclohept-1-ene (**S2**)*
^{1}H NMR spectrum S22
^{13}C NMR spectrum S22
*(7RS,4R)-1-(3-Iodobut-3-en-1-yl)-7-methyl-4-(prop-1-en-2-yl)cyclohept-1-ene (**S3**)*
^{1}H NMR spectrum S23
^{13}C NMR spectrum S23

Optimisation of the butenylation reaction leading to ketone 18

*Table S1. Direct butenylation of enone **14*** S24
*Table S2. Direct butenylation of enone **13*** S24
*Table S3. Alkylation of enone **14** with 1,4-dihalobut-2-ene* S24
*Table S4. Reductive rearrangement of ketone **15*** S25

^{1}H NMR (400 MHz, $CDCl_3$) [*dr* ~ 3:1]

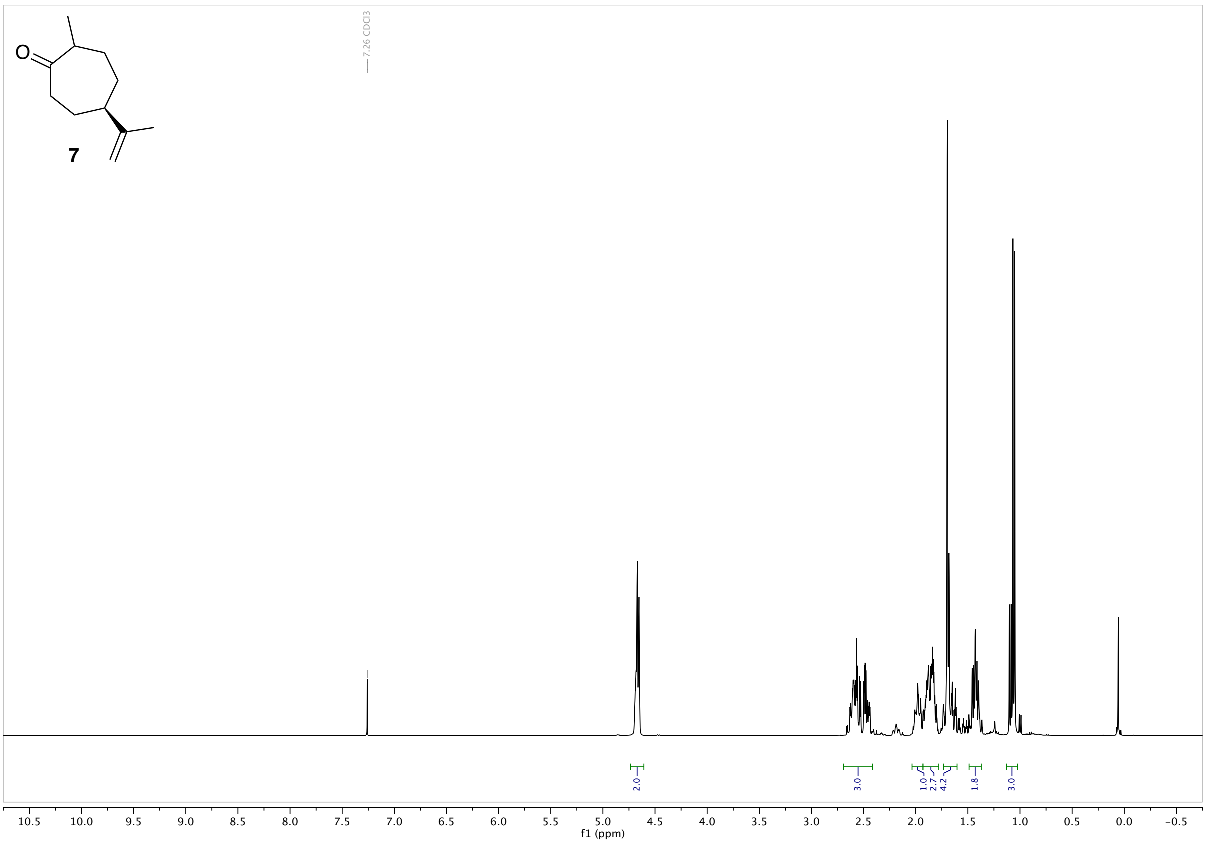

^{13}C NMR (101 MHz, $CDCl_3$) [*dr* ~ 3:1]

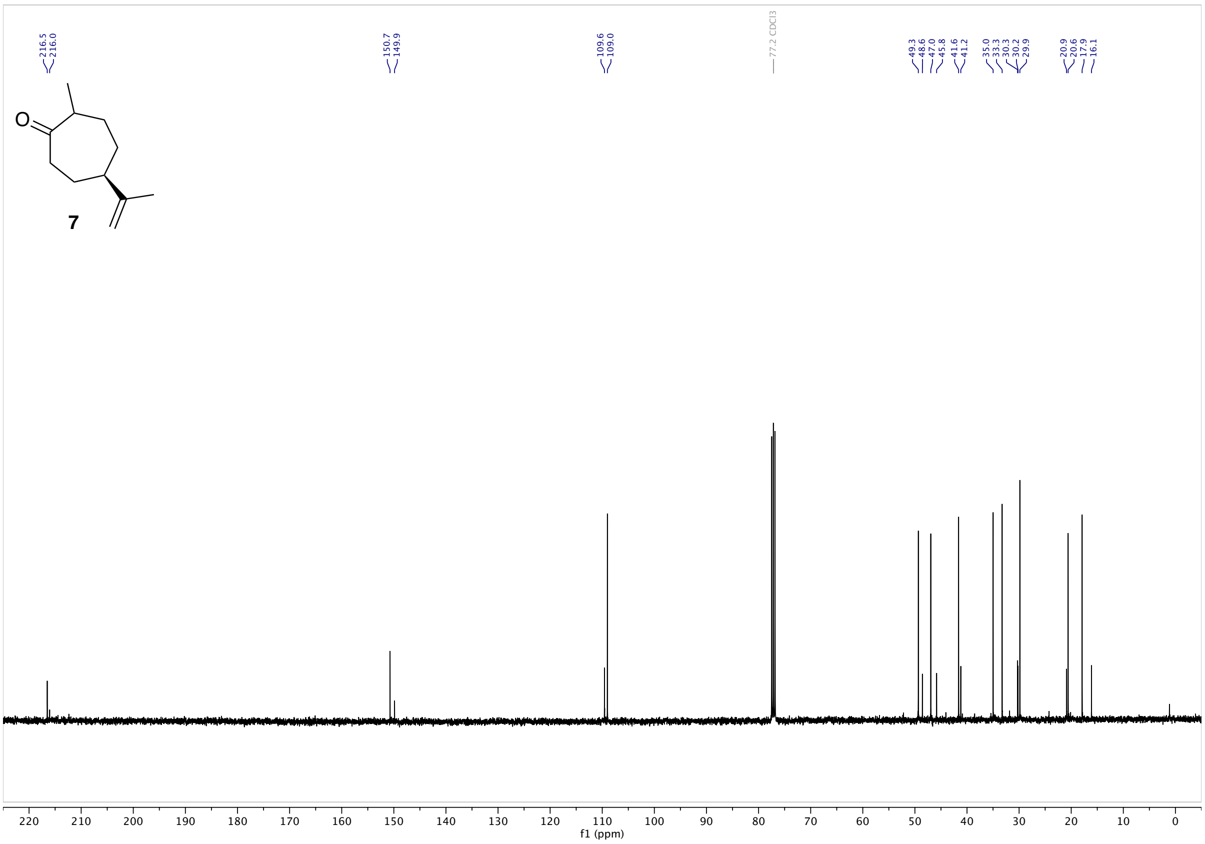

^{1}H NMR (400 MHz, CDCl$_3$)

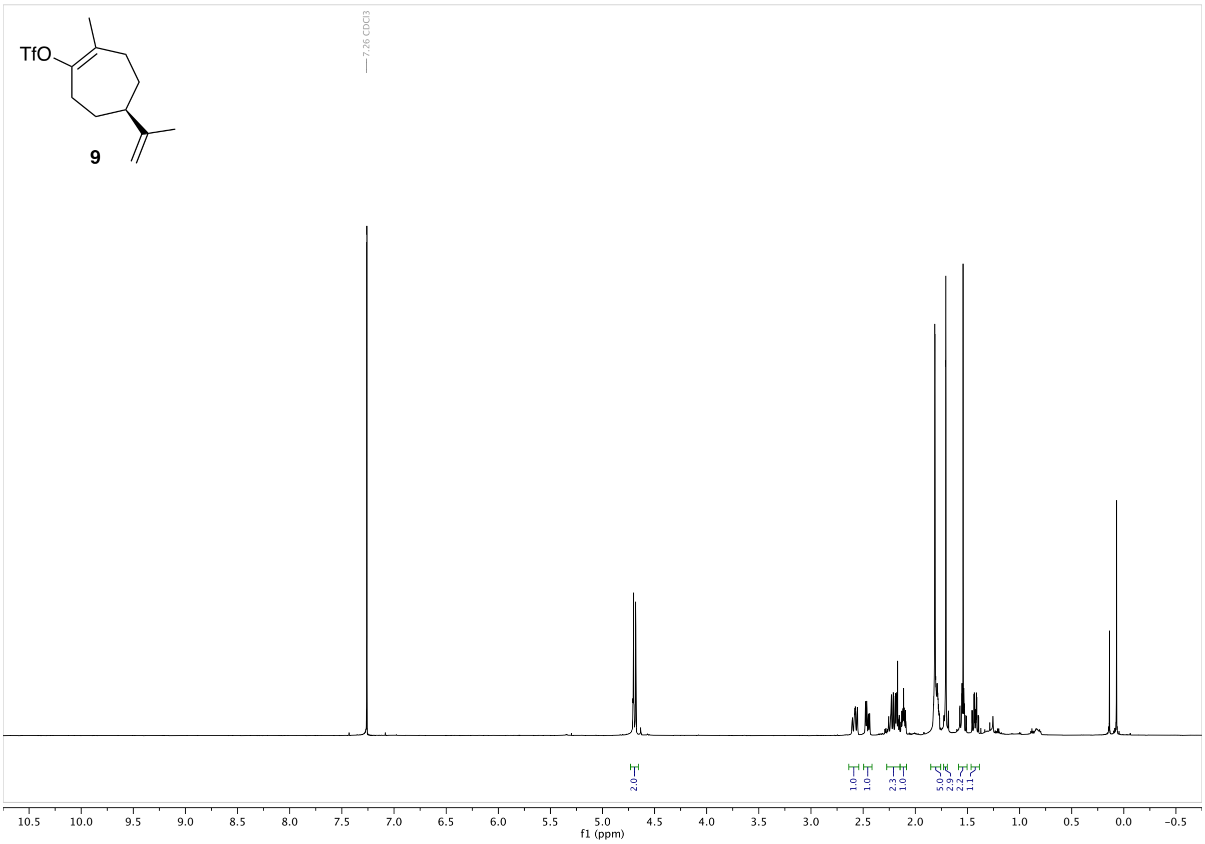

^{13}C NMR (101 MHz, CDCl$_3$)

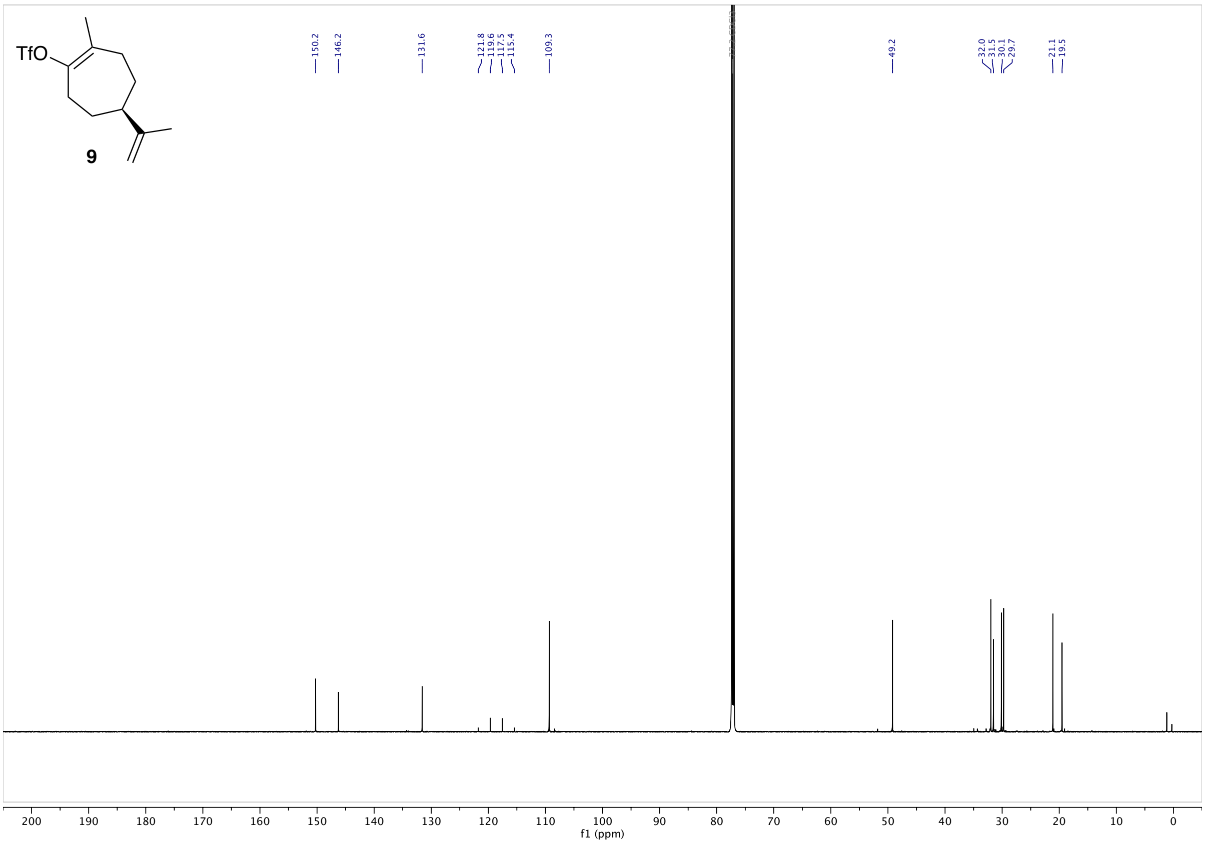

^{1}H NMR (400 MHz, $CDCl_3$)

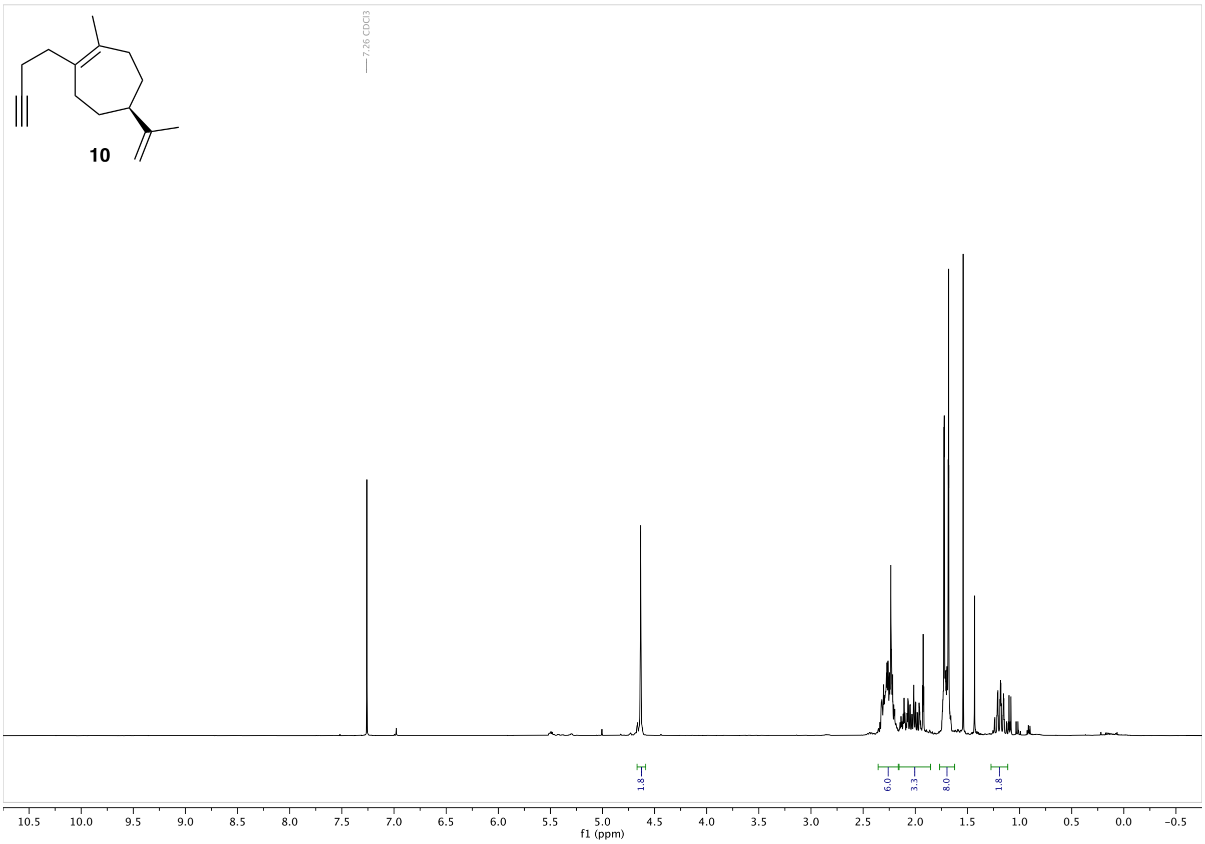

^{13}C NMR (101 MHz, $CDCl_3$)

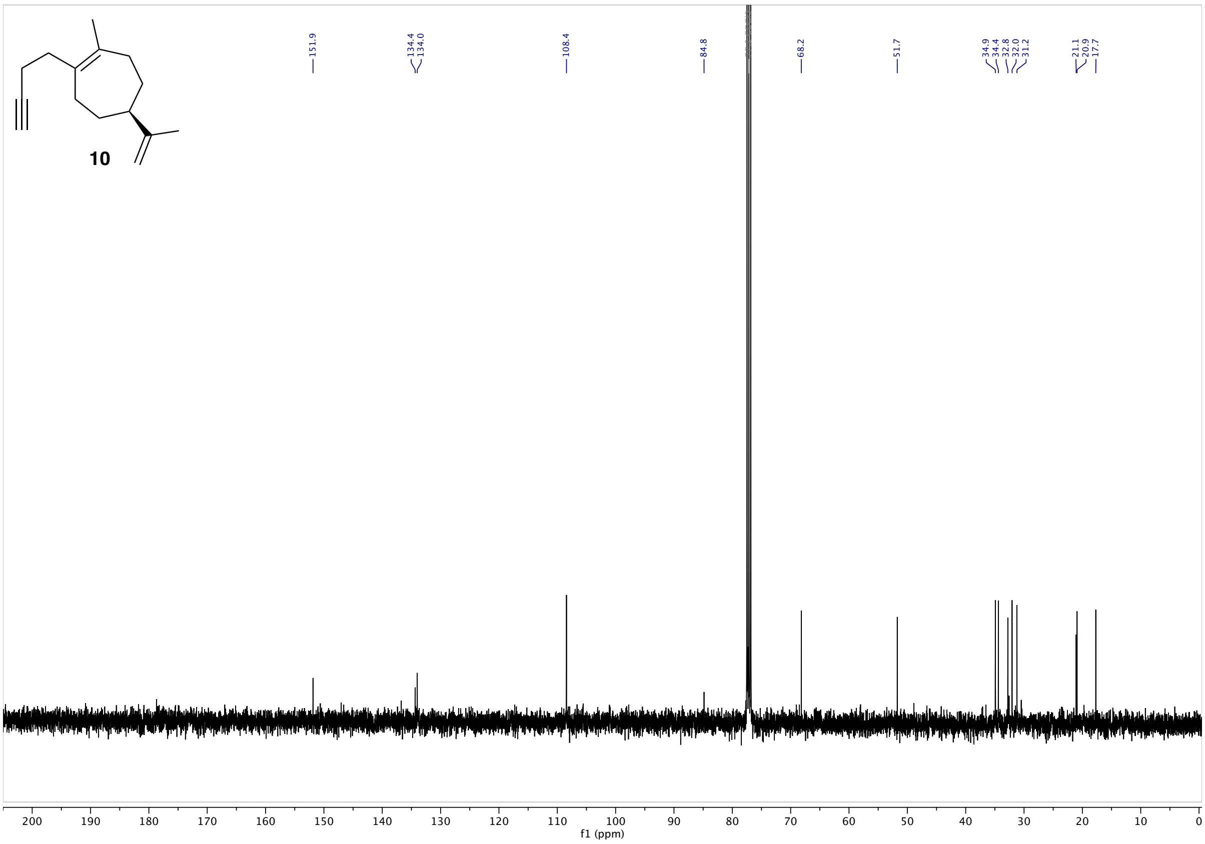

^{1}H NMR (400 MHz, $CDCl_3$)

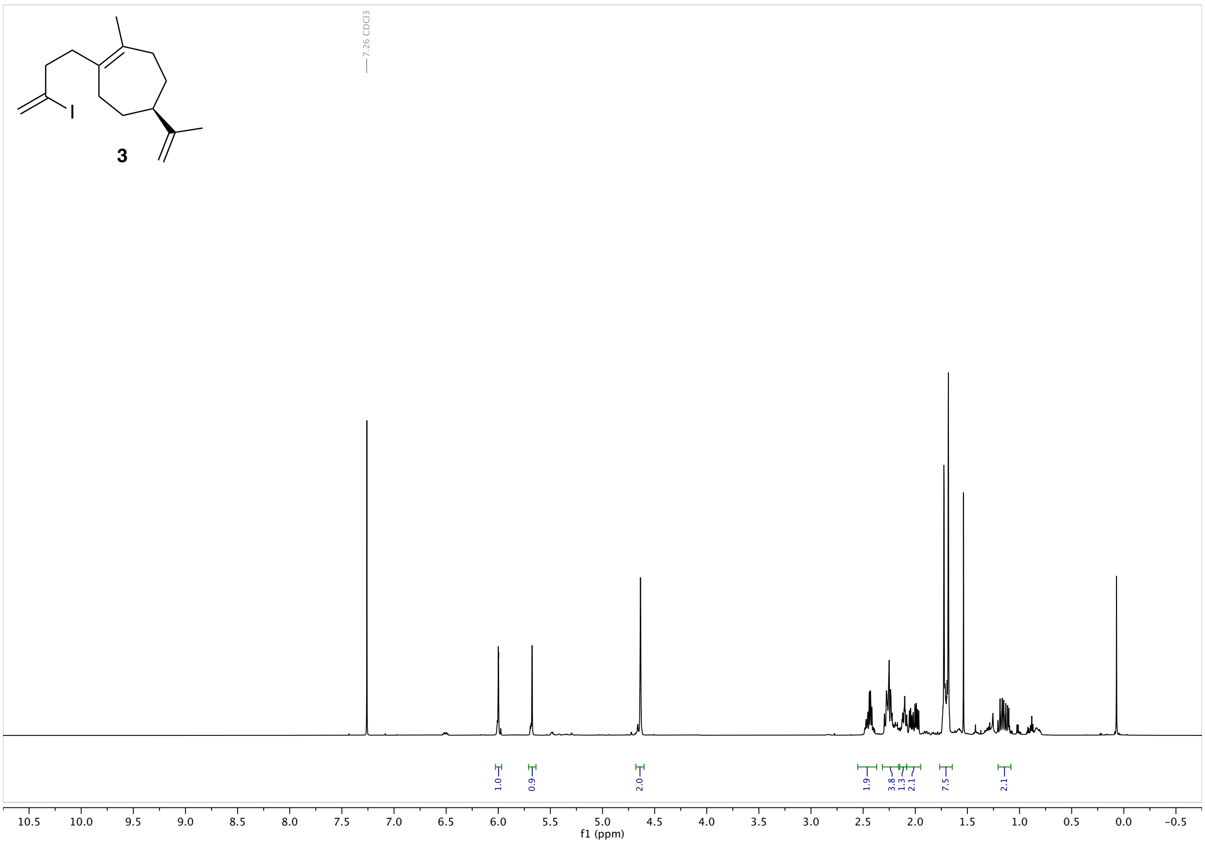

^{13}C NMR (101 MHz, $CDCl_3$)

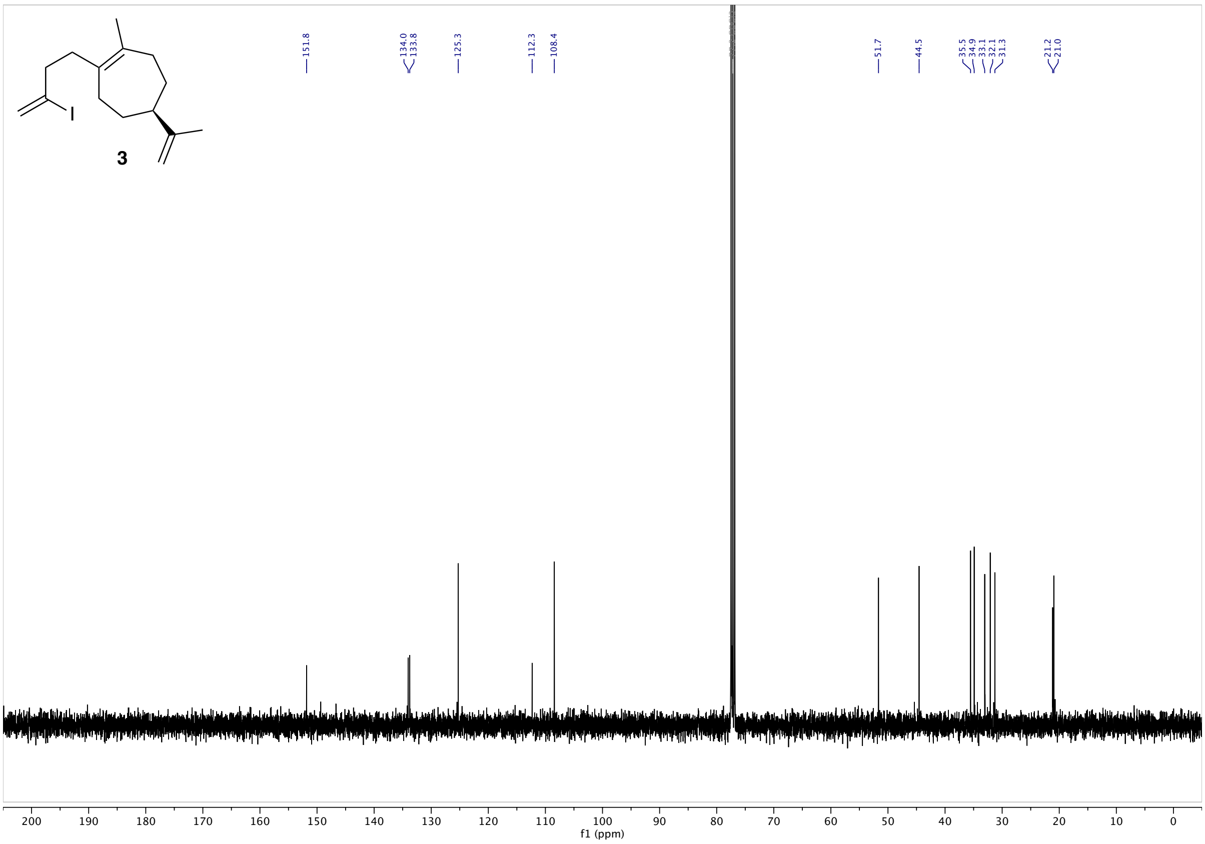

^{1}H NMR (400 MHz, $CDCl_3$) [*dr* ~ 1:1]

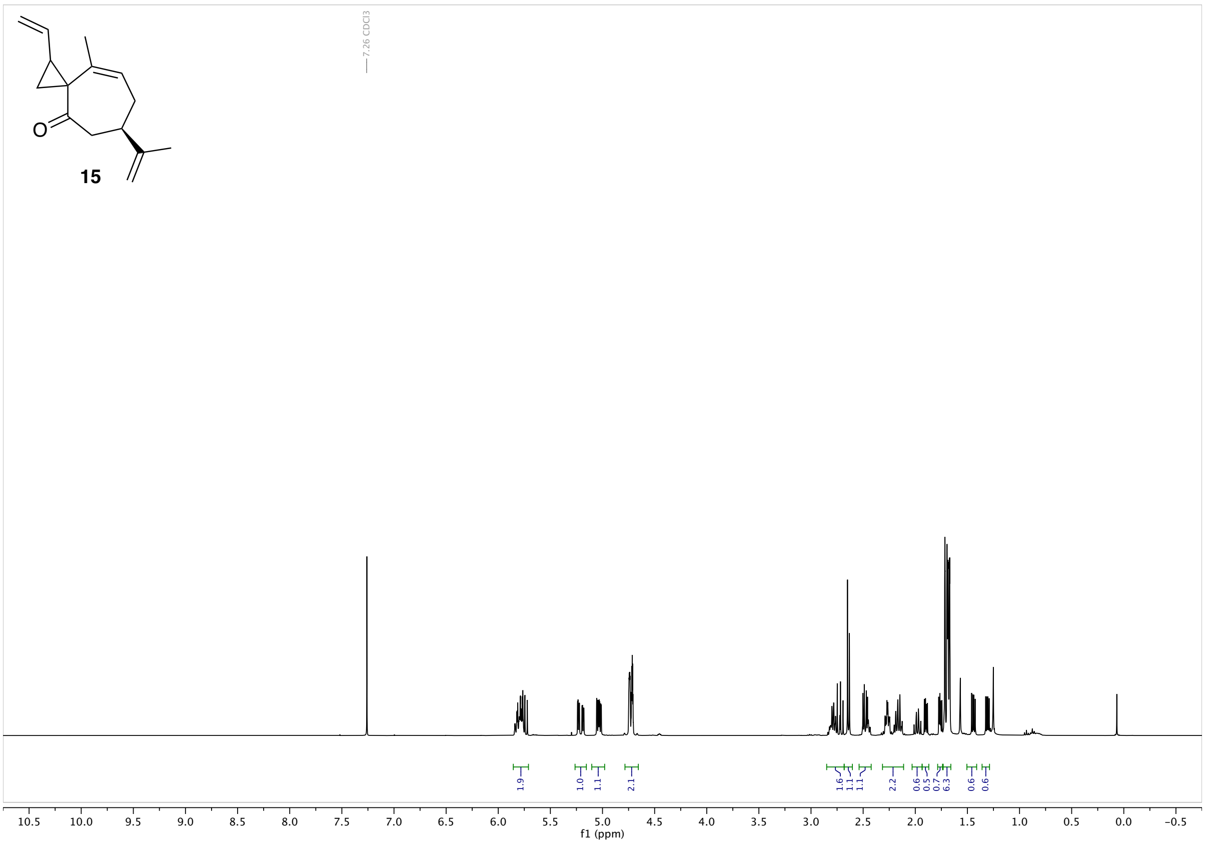

^{13}C NMR (101 MHz, $CDCl_3$) [*dr* ~ 1:1]

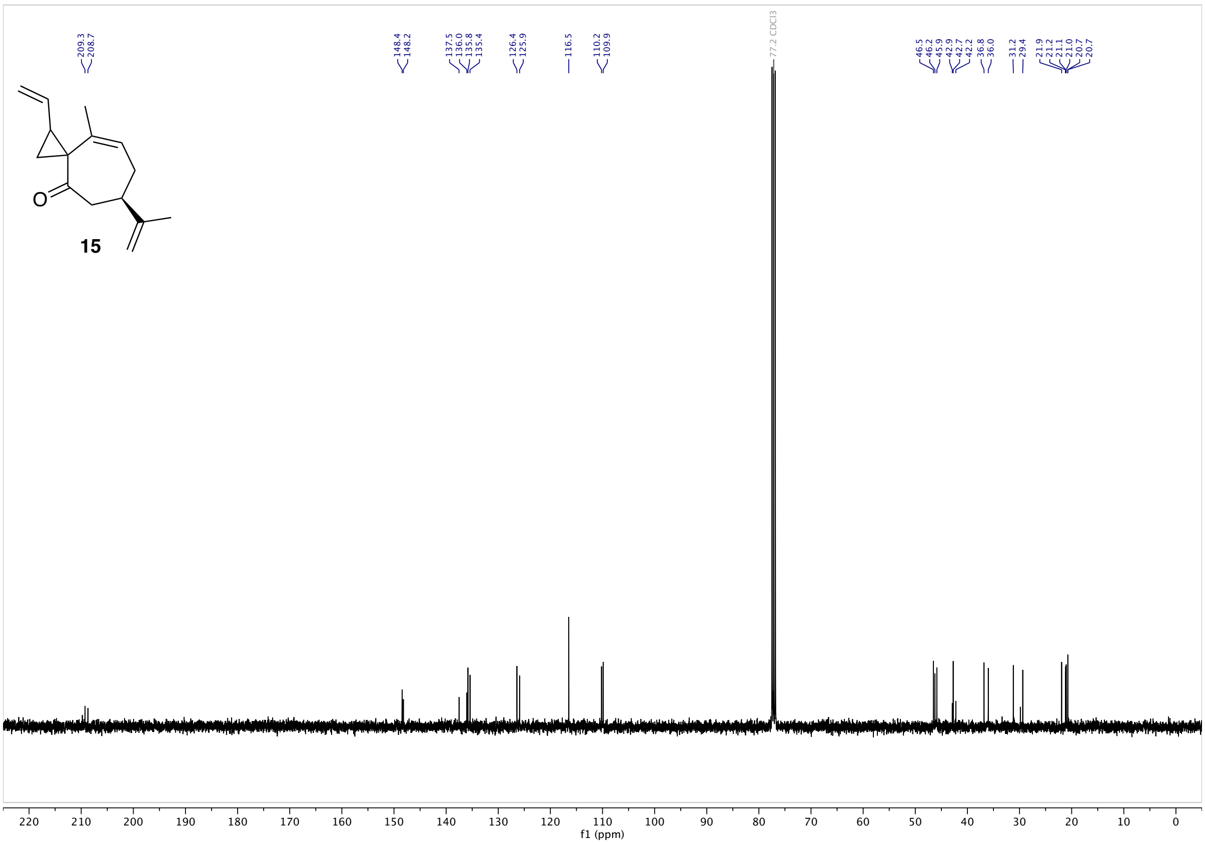

^{1}H NMR (400 MHz, $CDCl_3$) [*dr* ~ 2:1]

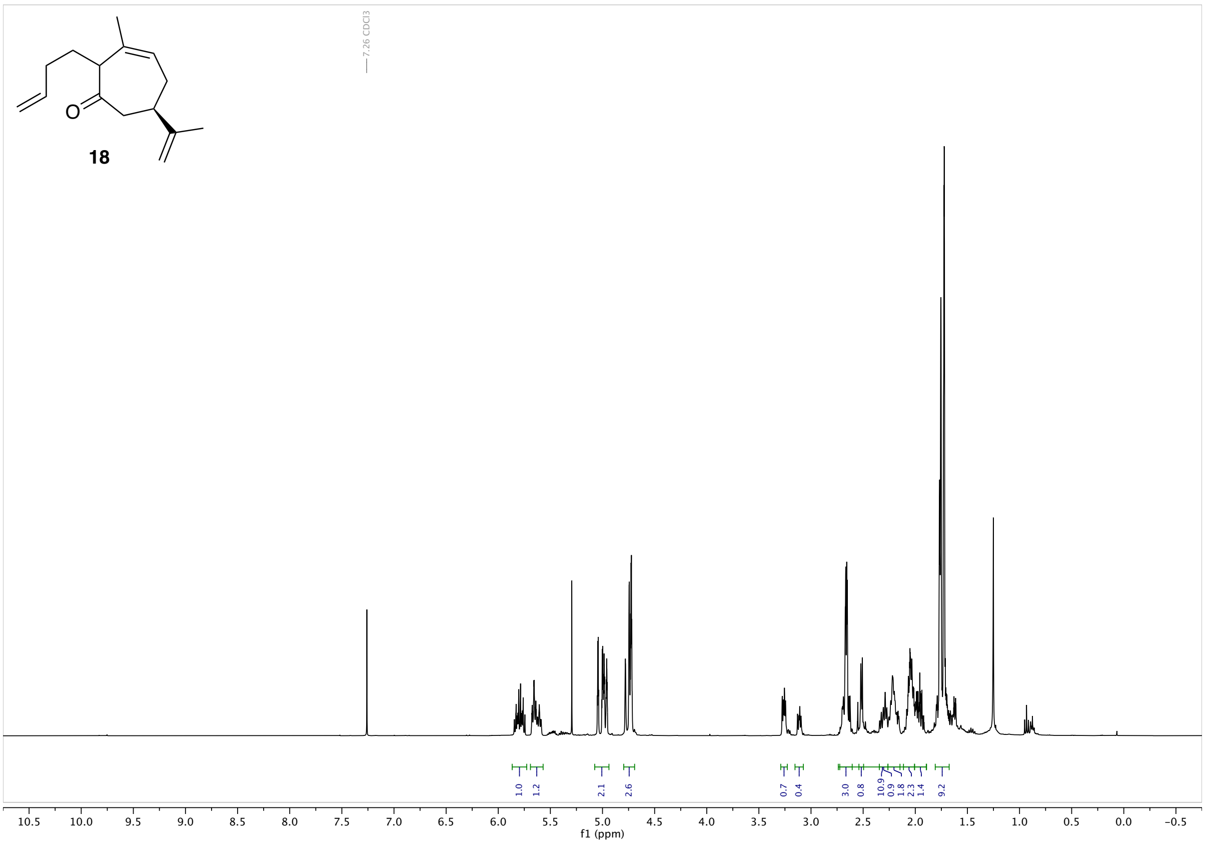

^{13}C NMR (101 MHz, $CDCl_3$) [*dr* ~ 2:1]

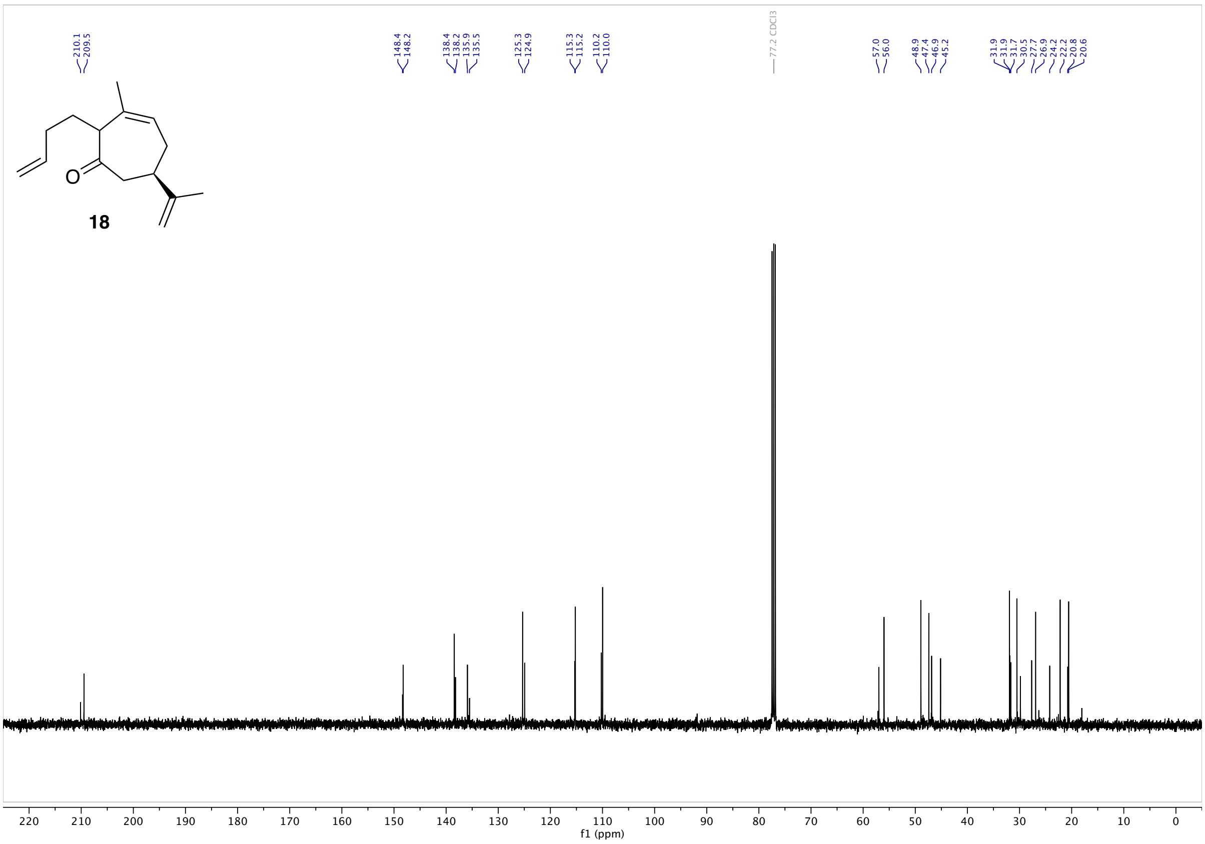

^{1}H NMR (400 MHz, $CDCl_3$)

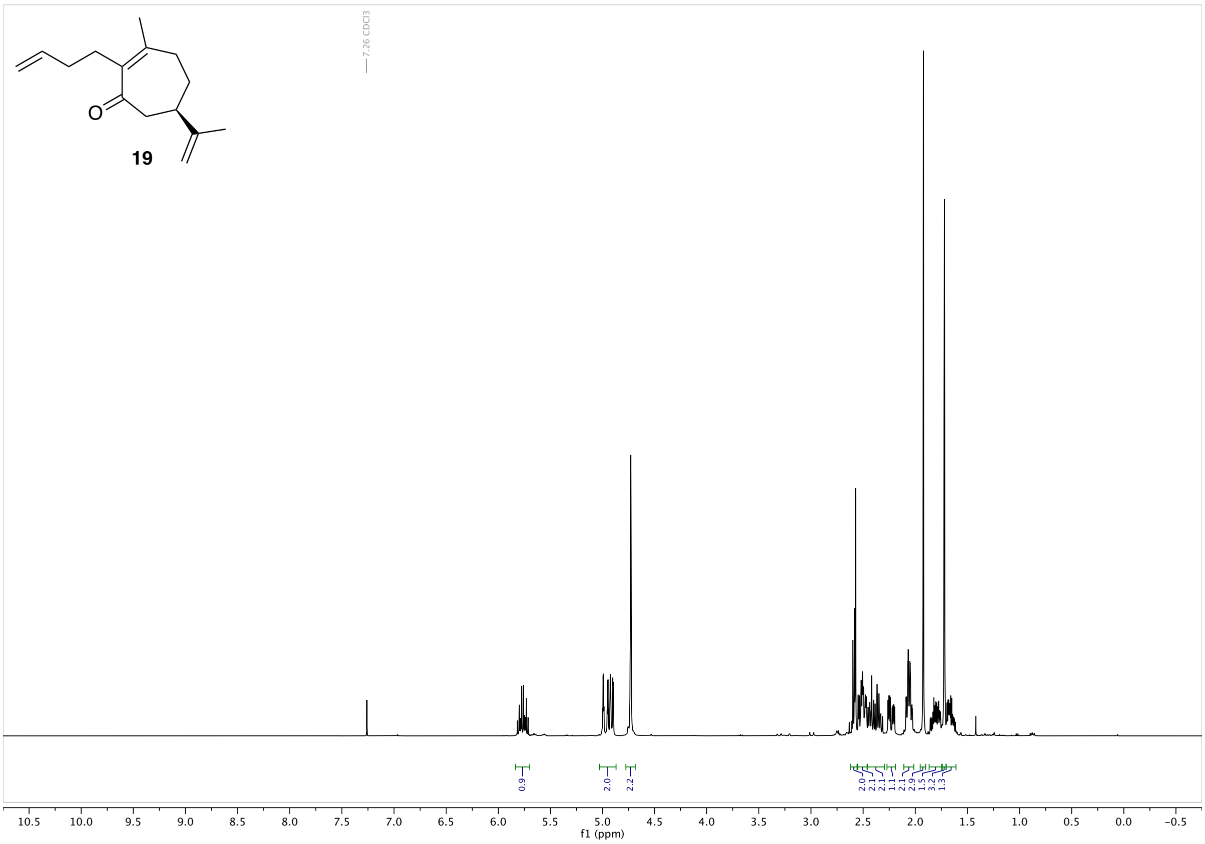

^{13}C NMR (101 MHz, $CDCl_3$)

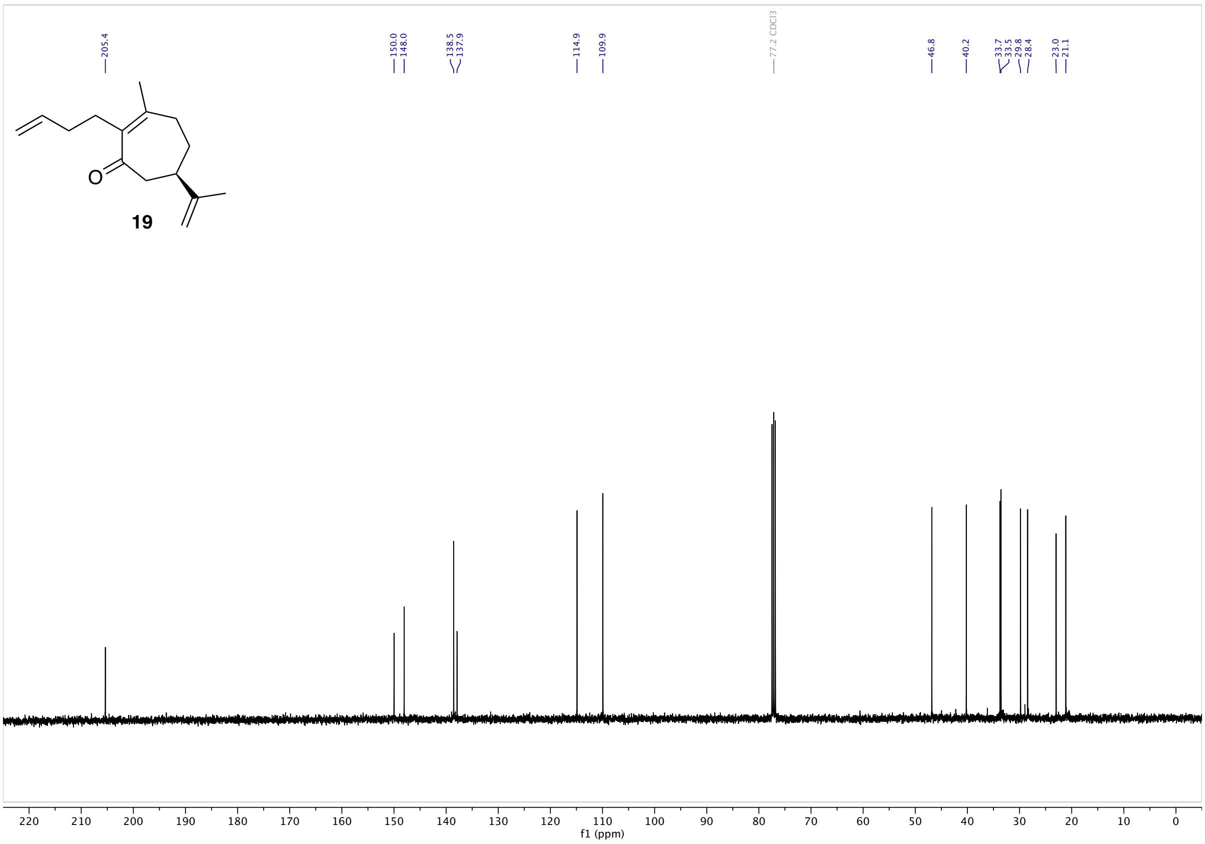

^{1}H NMR (600 MHz, C_6D_6) [*dr* ~ 5:1]

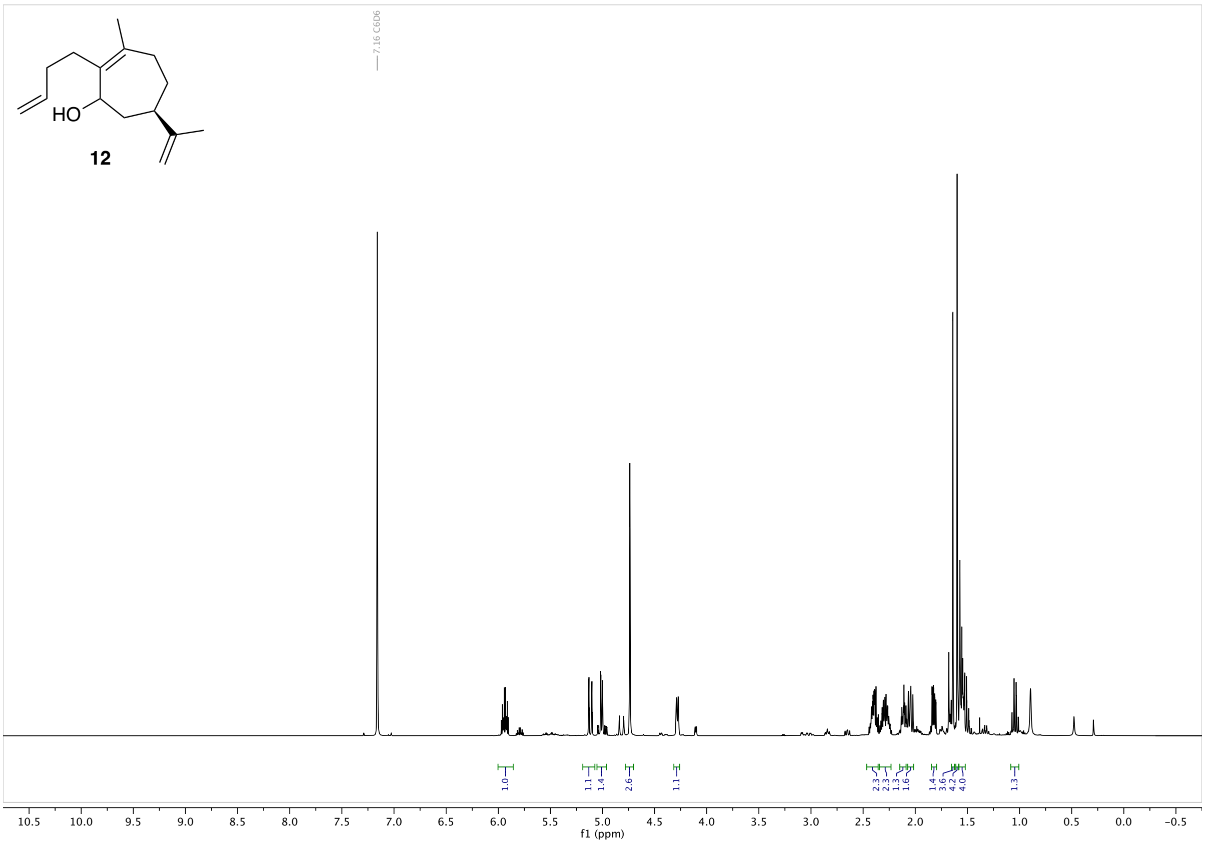

^{13}C NMR (151 MHz, C_6D_6) [*dr* ~ 5:1]

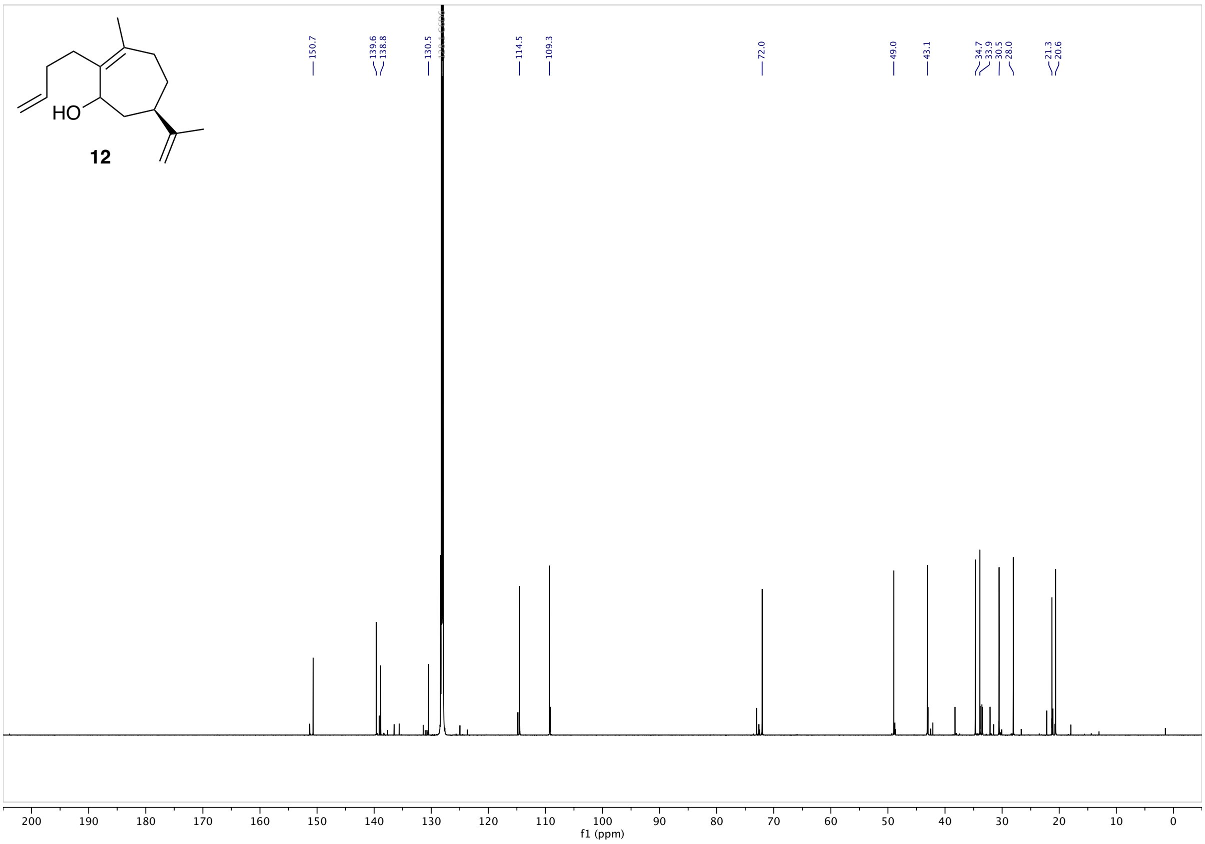

^{1}H NMR (600 MHz, acetone-d_6)

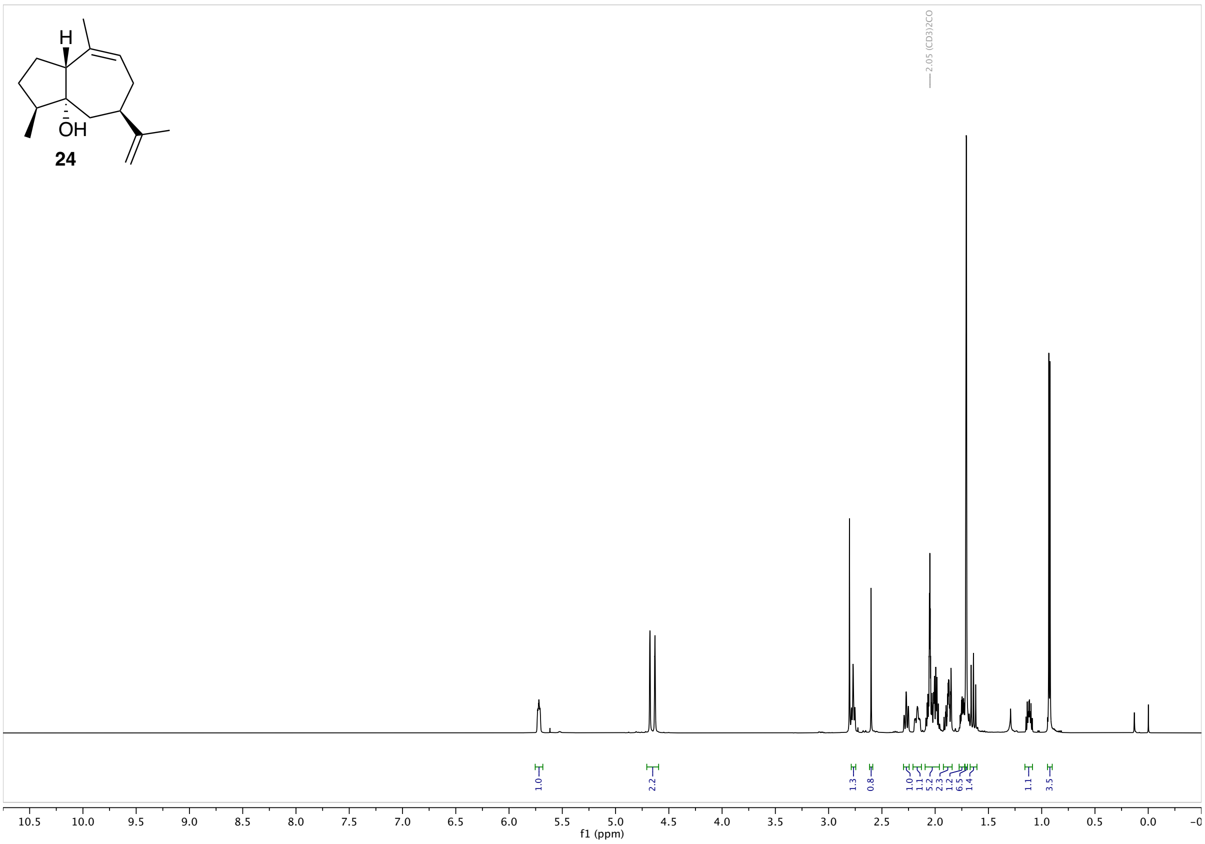

^{13}C NMR (151 MHz, acetone-d_6)

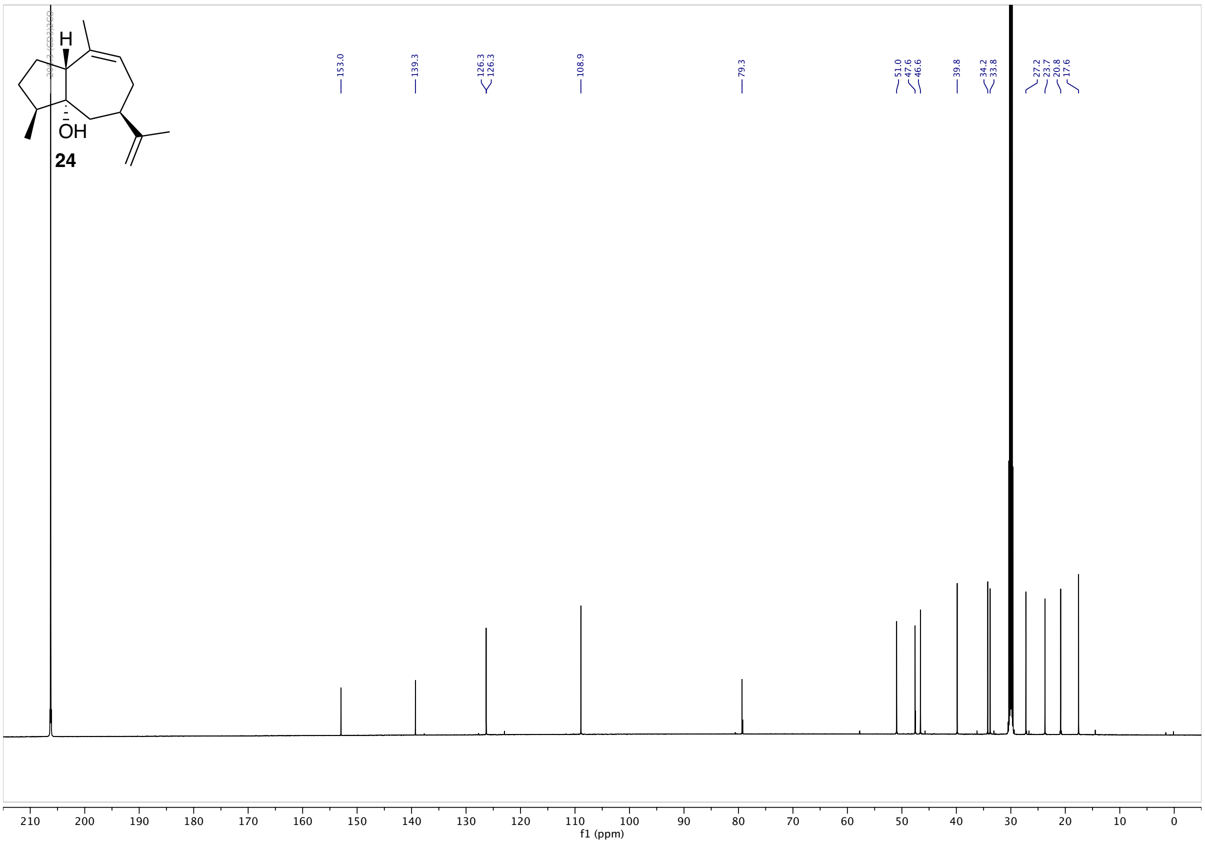

HSQC (acetone-d_6)

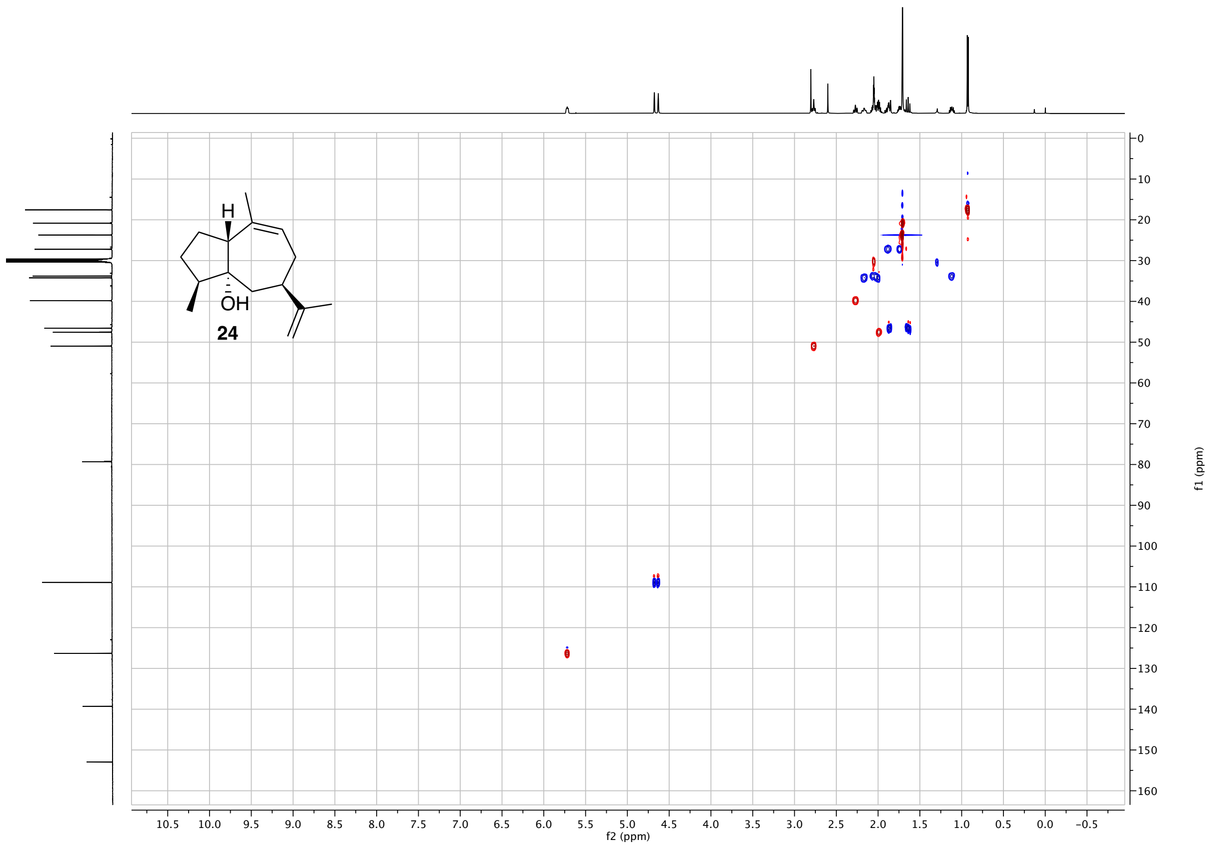

NOESY (acetone-d_6)

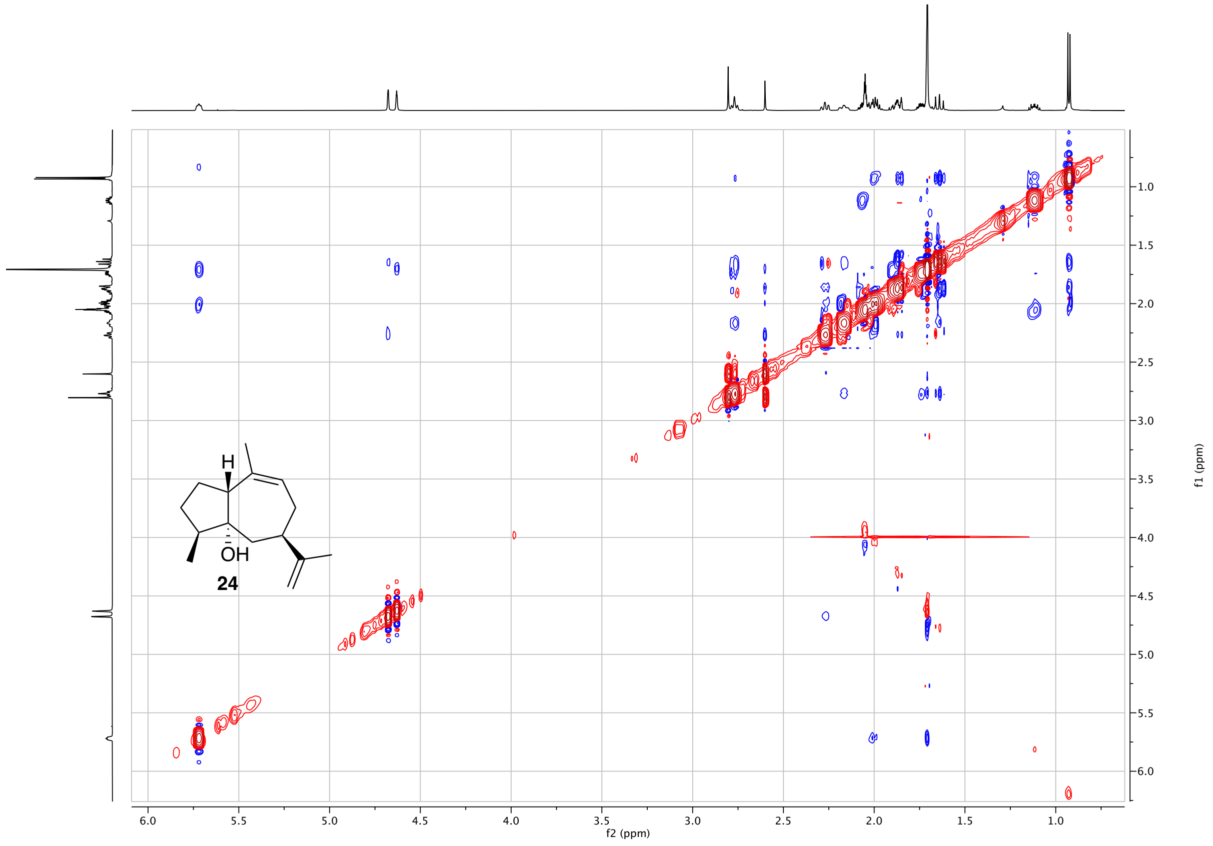

^{1}H NMR (600 MHz, acetone-d_6)

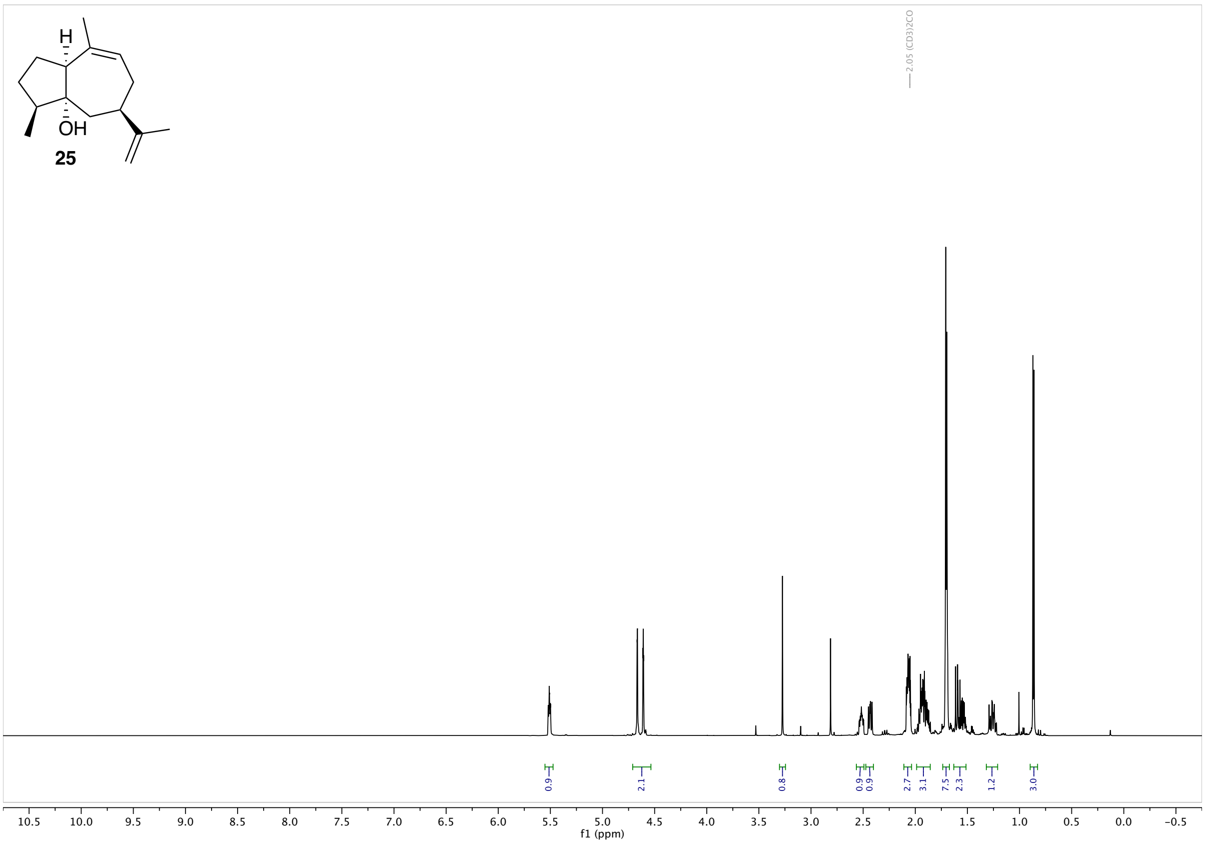

^{13}C NMR (151 MHz, acetone-d_6)

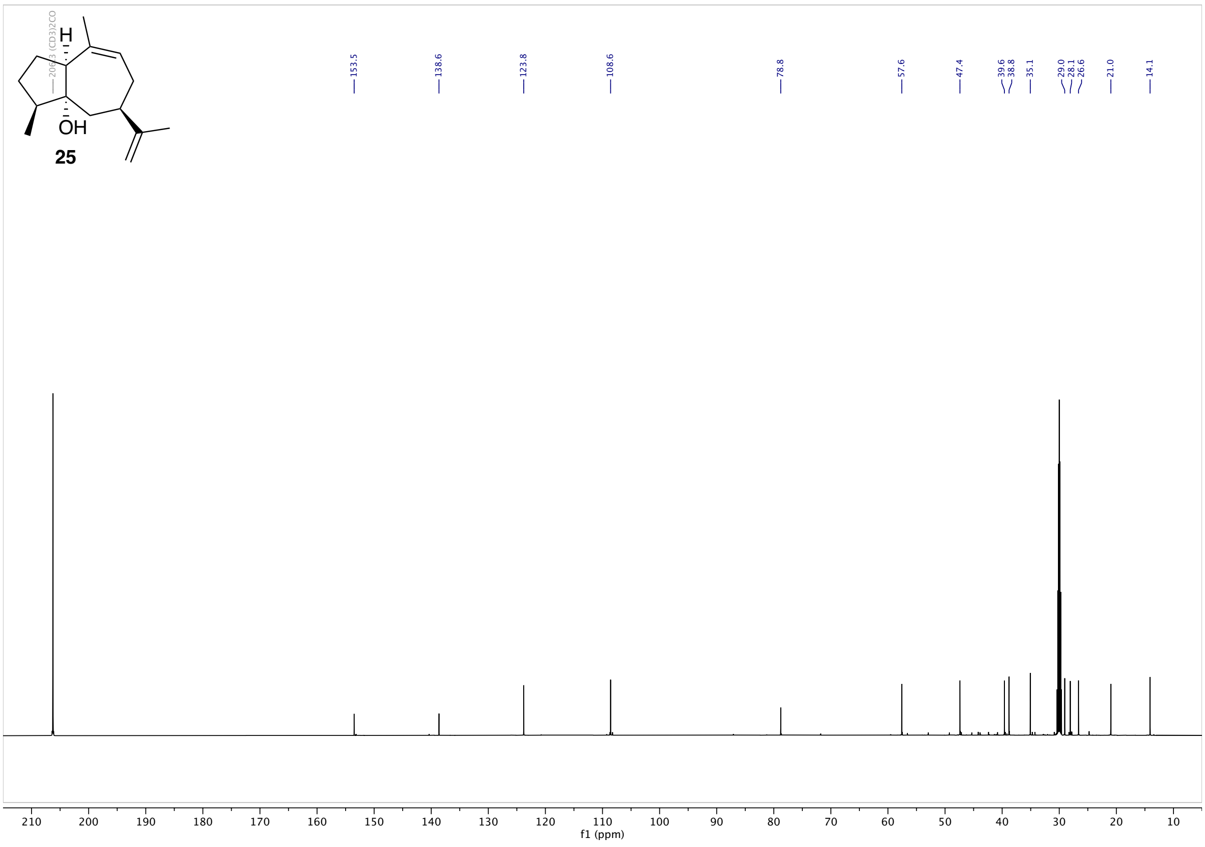

HSQC (acetone-d_6)

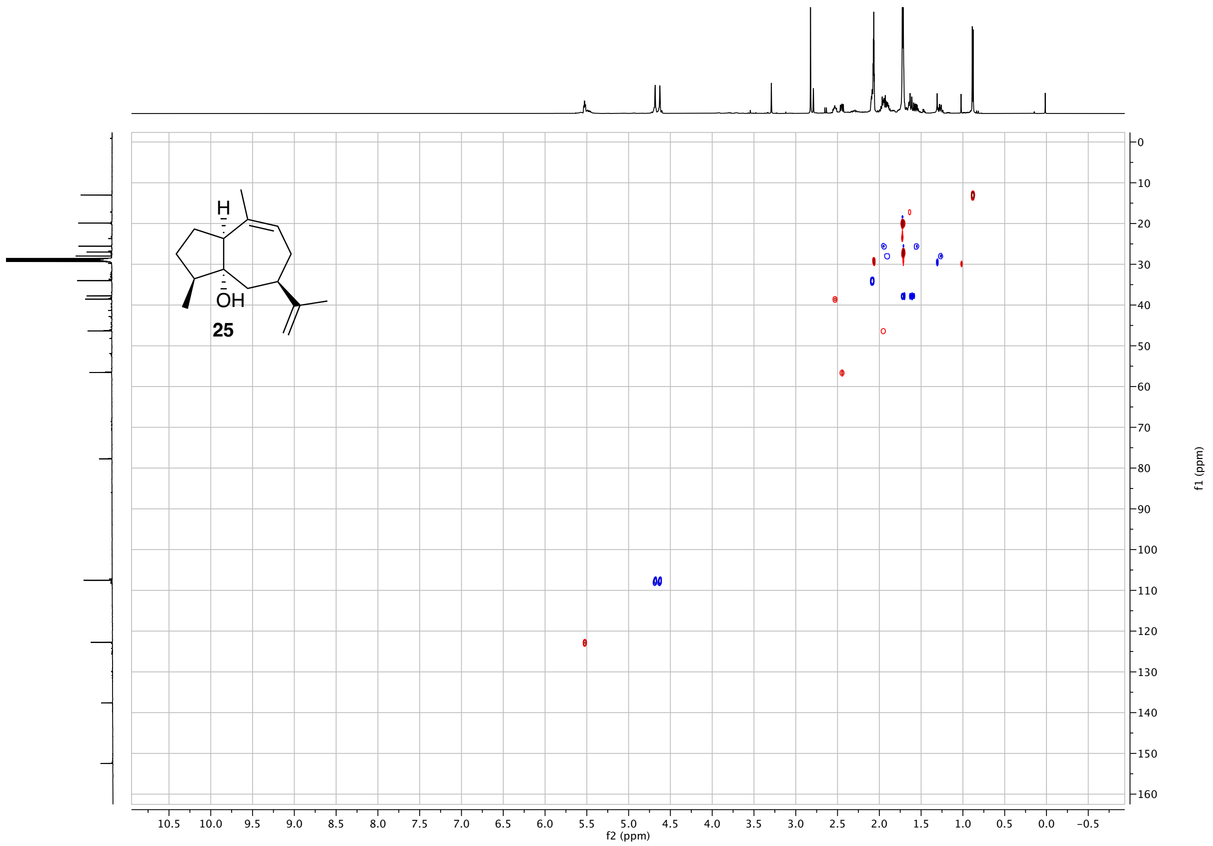

NOESY (acetone-d_6)

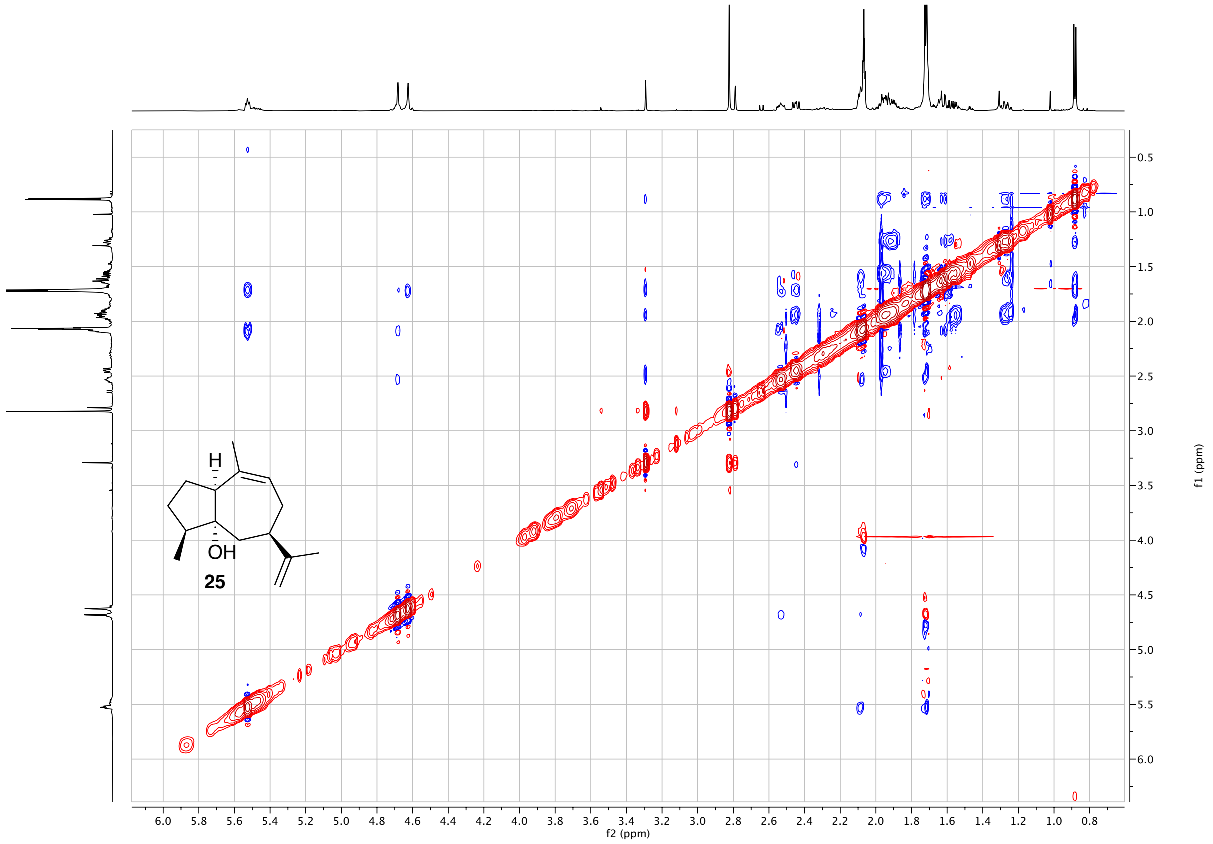

^1H NMR (600 MHz, $CDCl_3$)

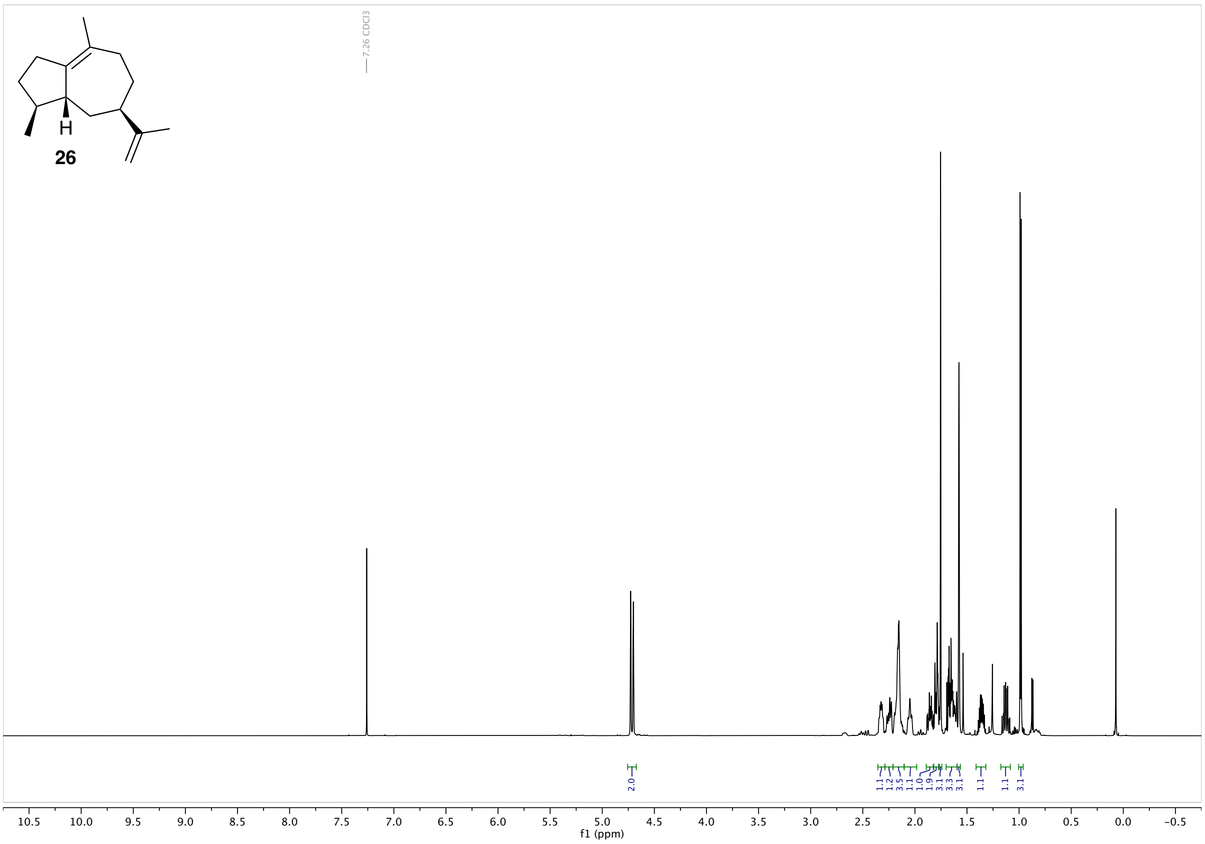

^{13}C NMR (151 MHz, $CDCl_3$)

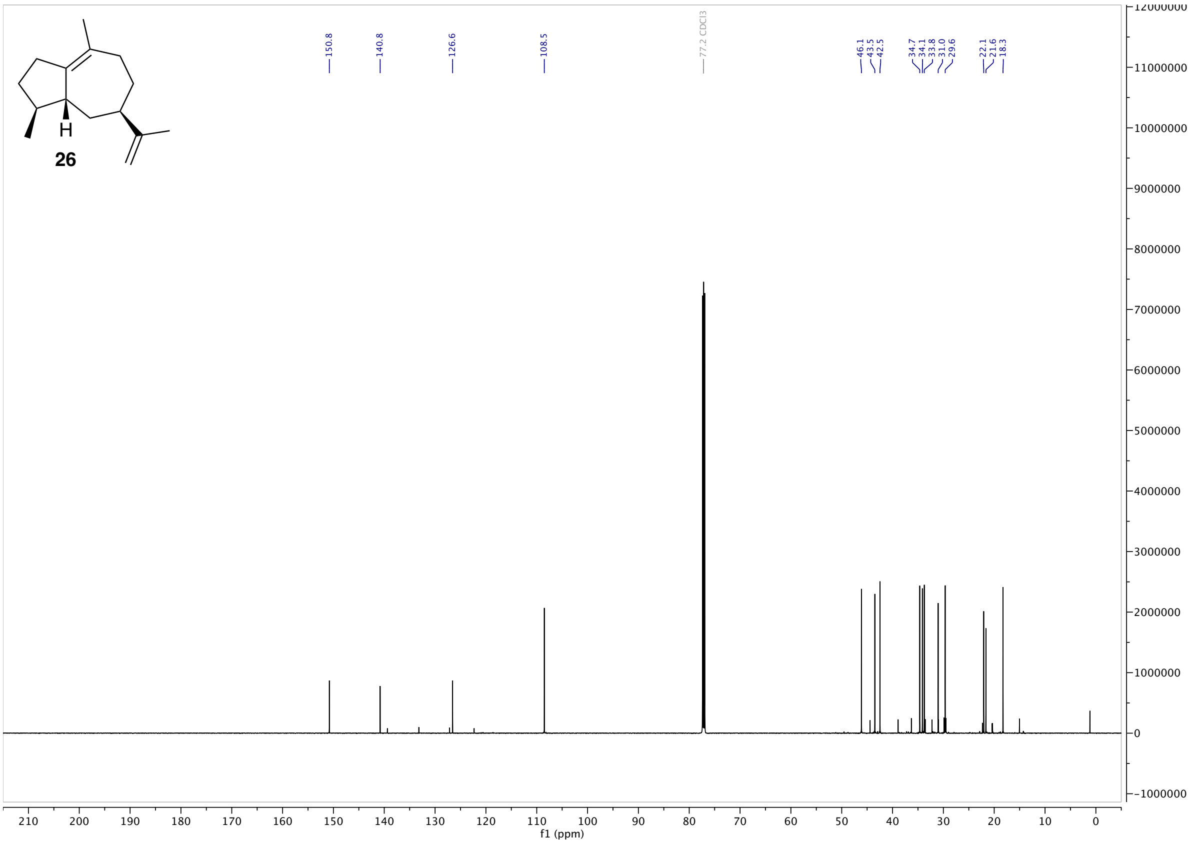

HSQC ($CDCl_3$)

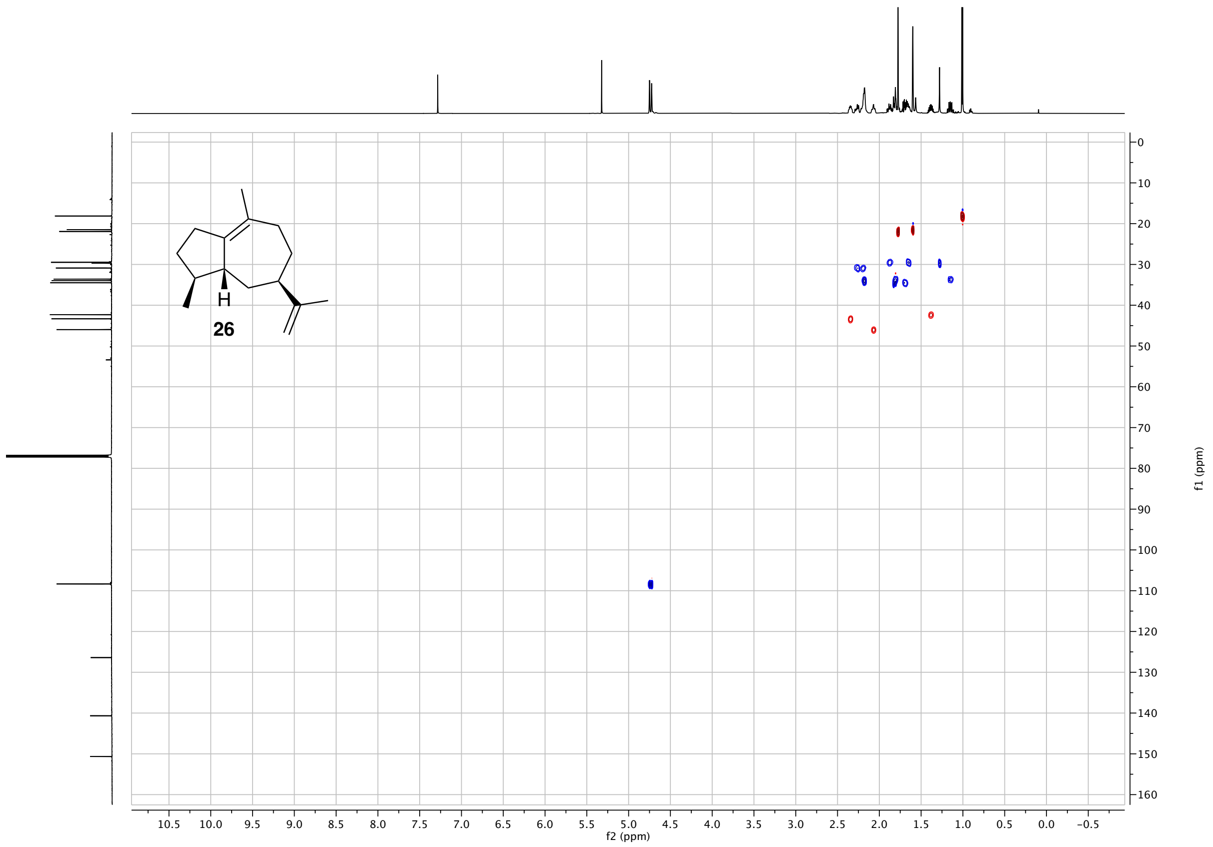

HMBC ($CDCl_3$)

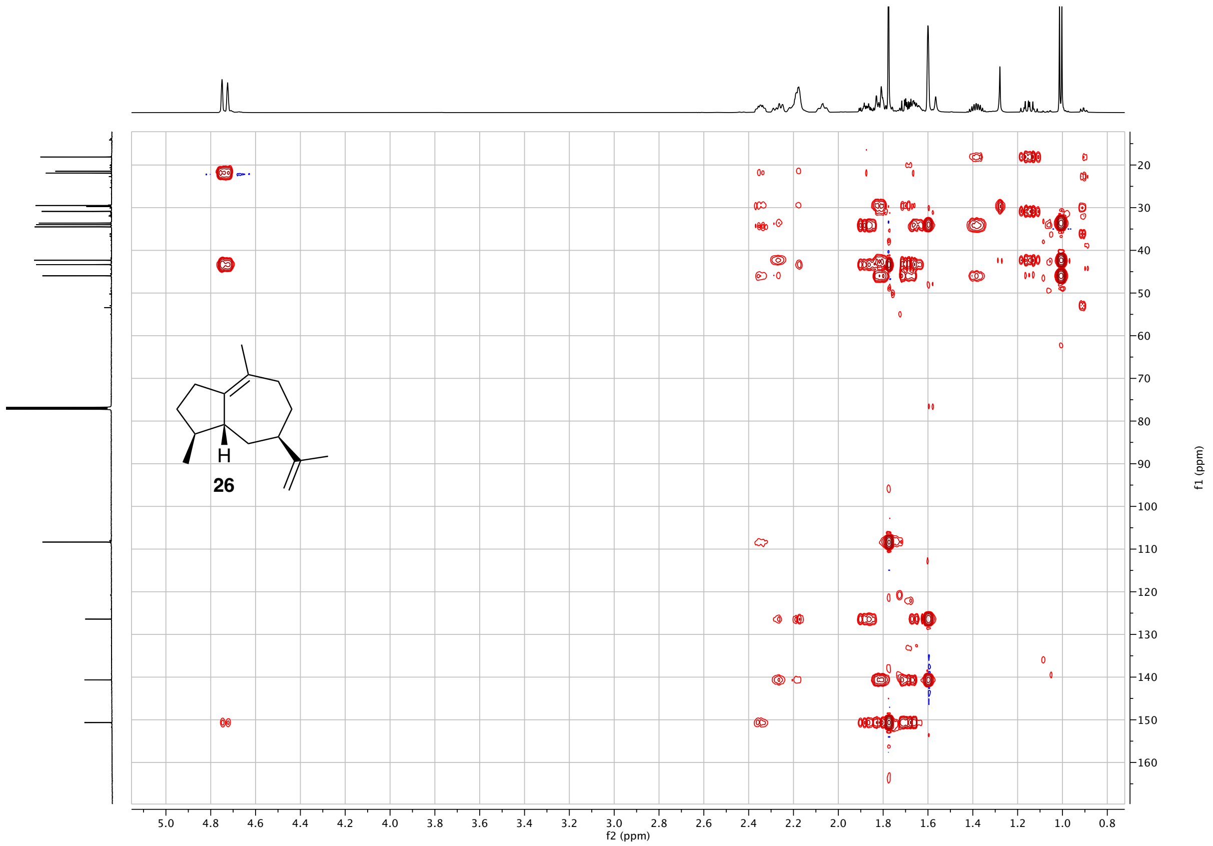

NOESY ($CDCl_3$)

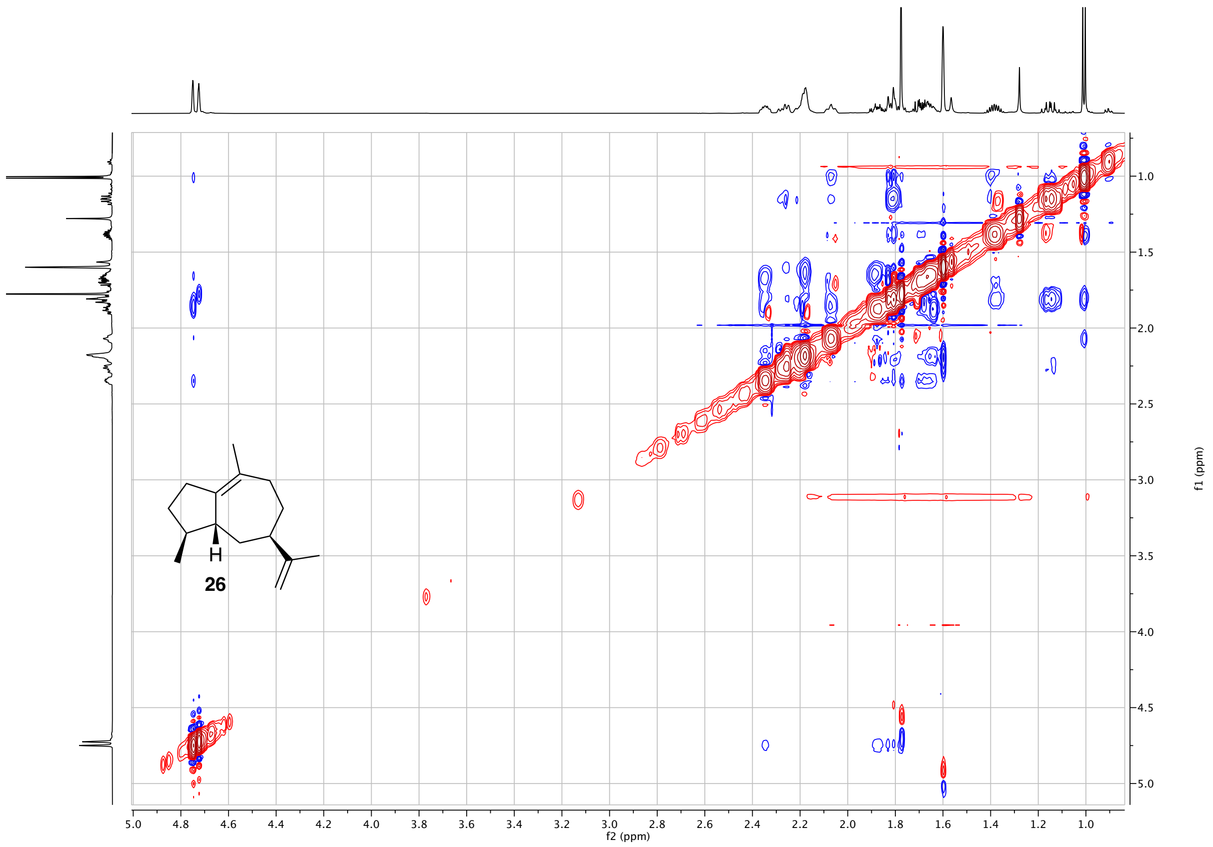

^{1}H NMR (600 MHz, $CDCl_3$)

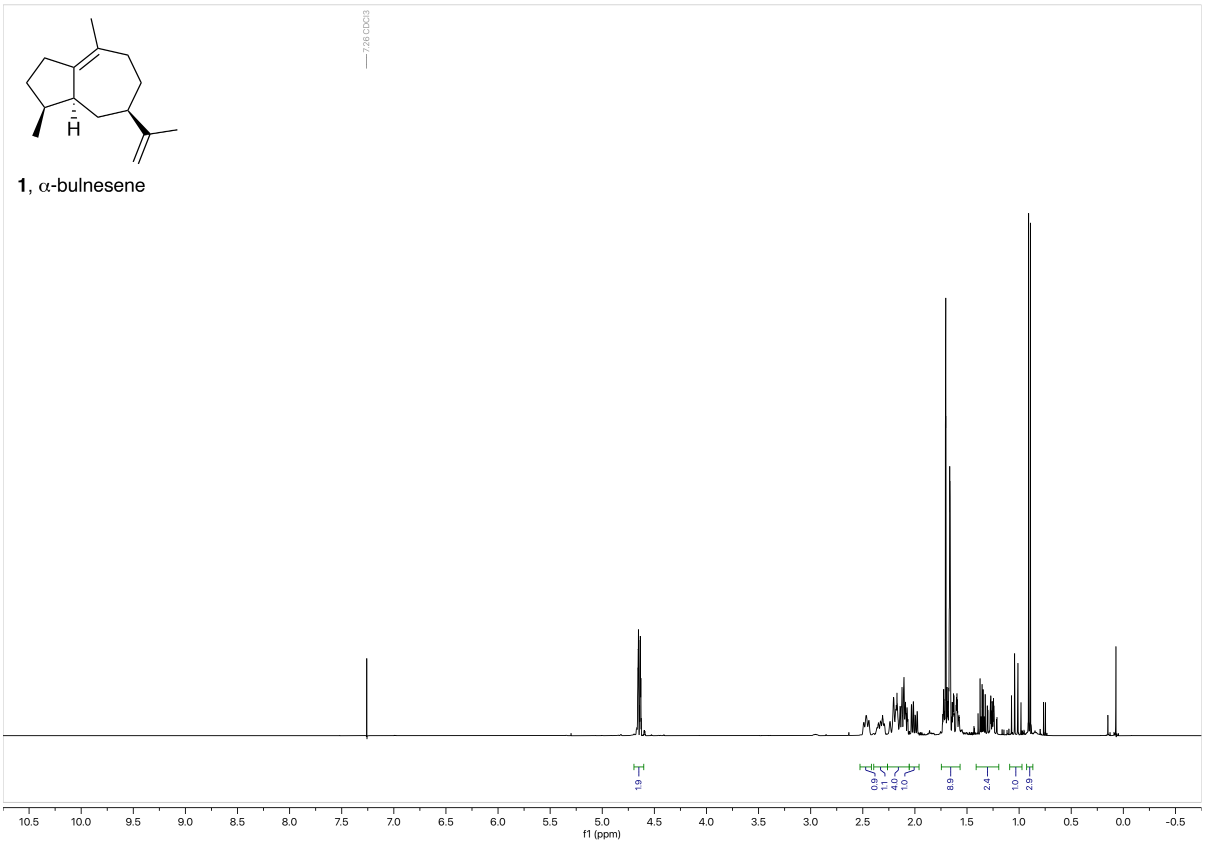

^{13}C NMR (151 MHz, $CDCl_3$)

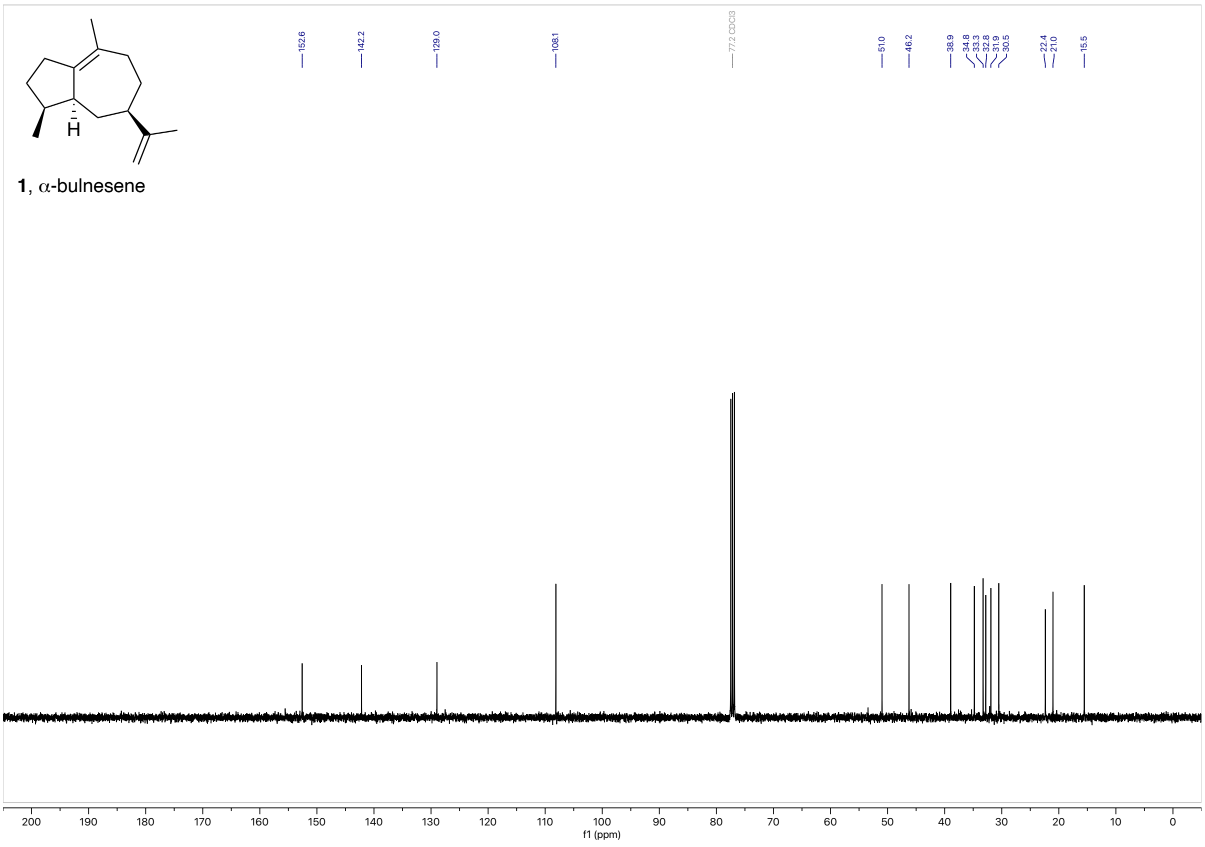

Alkene regioisomers of compounds 9, 10, and 13

*(7RS,4R)-7-Methyl-4-(prop-1-en-2-yl)cyclohept-1-en-1-yl trifluoromethanesulfonate (**S1**)*

LHMDS (2.4 mL, 1.0 M in THF, 2.4 mmol) was added dropwise to a stirred solution of ketone **7** (332 mg, 2.00 mmol) in dry THF (10 mL) at –78 °C. The solution was stirred at this temperature for 1 h then warmed to RT and stirred for another 2 h. A solution of Comins' reagent (1.10 g, 2.80 mmol) in dry THF (5 mL) was added and the mixture was stirred at RT for 14 h. The mixture was diluted with ether (20 mL) and washed with saturated aqueous NH_4Cl solution (10 mL) then brine (20 mL). The organic layer was dried ($MgSO_4$), filtered, and concentrated. The residue was purified by column chromatography (pentane/ethyl acetate, 1000:1) to give the title compound (**S1**), a colourless oil (485 mg, 81%), as a 2.8:1 ratio of diastereomers. R_f 0.28 (pentane); ν_{max}/cm^{-1} 2941m, 1413m, 1141m; δ_H (400 MHz, $CDCl_3$) [major diastereomer] 5.82 (1H, t, *J* = 7.0Hz), 4.69 (2H, s), 2.75–2.63 (1H, m), 2.39–2.30 (1H, m), 2.22 (2H, app t, *J* = 6.5 Hz), 2.00–1.87 (2H, m), 1.70 (3H, s), 1.64–1.53 (2H, m), 1.21 (3H, d, *J* = 7.0 Hz); δ_C (101 MHz, $CDCl_3$) [major/minor] 155.7/156.4, 149.7/150.3, 120.4/120.5, 118.7 (q, *J* = 320 Hz (major)), 109.7/109.4, 45.0/45.5, 37.8/38.1, 30.8/31.3, 30.5/30.1, 28.0/29.1, 20.4/20.6, 17.5/17.1.

*(7RS,4R)-1-(But-3-yn-1-yl)-7-methyl-4-(prop-1-en-2-yl)cyclohept-1-ene (**S2**)*

A solution of [4-(trimethylsilyl)but-3-yn-1-yl]magnesium bromide (0.420 mmol) (prepared as in the procedure for the preparation of compound **10**) in THF (6 mL) was added rapidly to a –30 °C solution of enol triflate **S1** (63 mg, 0.21 mmol), $Fe(acac)_3$ (7.5 mg, 0.021 mmol), and NMP (0.25 mL) in dry THF (5 mL) under N_2, causing an immediate colour change from orange to black. The mixture was stirred for 15 min and was then quenched with saturated aqueous NH_4Cl solution (5 mL). The aqueous phase was extracted with ether (3 × 20 mL), the combined organic layers were dried (Na_2SO_4), and the solvent was evaporated. The residue was purified by column chromatography (pentane/ethyl acetate, 1000:1) to give the intermediate trimethylsilylalkyne as a colourless oil (44 mg, 76%) and as a 2.6:1 ratio of diastereomers. In a separate reaction, TBAF (2.4 mL, 1.0 M in THF, 2.4 mmol) was added into a solution of this intermediate (330 mg, 1.20 mmol) in dry THF (5 mL) and the mixture was stirred for 2 h at RT. The reaction was quenched with saturated aqueous NH_4Cl solution (10 mL) and the aqueous layer was extracted with ether (3 × 10 mL). The combined organic layers were dried ($MgSO_4$), filtered, and concentrated. The residue was purified by column chromatography (pentane/ethyl acetate, 1000:1) to give the title compound (**S2**), a colourless oil (130 mg, 54%), as a 5.5:1 ratio of diastereomers. R_f 0.31 (pentane); ν_{max}/cm^{-1} 3216m; δ_H (400 MHz, $CDCl_3$) [major diastereomer] 5.52–5.47 (1H, m), 4.68–4.65 (1H, m), 4.64–4.62 (1H, m), 2.49–2.38 (1H, m), 2.30–2.24 (2H, m), 2.24–2.08 (5H, m,), 1.96 (1H, t, *J* = 2.5 Hz), 1.93–1.84 (1H, m), 1.78–1.68 (1H, m) overlaying 1.70 (3H, s), 1.64–1.54 (1H, m), 1.46–1.36 (1H, m), 1.09 (3H, d, *J* = 7.0 Hz); δ_C (101 MHz, $CDCl_3$) [major diastereomer] 151.3, 145.2, 124.5, 108.6, 84.7, 68.5, 45.2, 36.5, 34.9, 32.7, 32.6, 31.5, 20.7, 18.9, 18.6.

*(7RS,4R)-1-(3-Iodobut-3-en-1-yl)-7-methyl-4-(prop-1-en-2-yl)cyclohept-1-ene (**S3**)*

DIBAL (0.83 mL, 1.2 M in toluene, 1.0 mmol) was added dropwise to a solution of $Ni(dppp)Cl_2$ (54 mg, 0.10 mmol) in dry THF (15 mL) under Ar. The mixture was cooled to 0 °C and alkyne **S2** (101 mg, 0.499 mmol) was added via syringe into the black solution; the mixture was warmed to RT and stirred for 4 h then cooled

to 0 °C. A solution of *N*-iodosuccinimide (338 mg, 1.50 mmol) in THF (5 mL) was added dropwise then the solution was warmed to RT and stirred for 18 h. The reaction was quenched with saturated aqueous Rochelle's salt solution (10 mL), the aqueous phase was extracted with ether (3 × 20 mL), and the combined organic extracts were dried ($MgSO_4$), filtered, and concentrated. The residue was purified by column chromatography (pentane/triethylamine, 100:1) to give vinyl iodide **S3** as a colourless oil (76 mg, 46%), isolated as a single (unassigned) diastereomer. R_f 0.50 (pentane); ν_{max}/cm^{-1} 2961m, 2929m, 889m; δ_H (400 MHz, $CDCl_3$) 6.01 (1H, q, *J* = 1.5 Hz), 5.68 (1H, app d, *J* = 1.5 Hz), 5.51–5.46 (1H, m), 4.67–4.61 (2H, m), 2.47 (2H, t, *J* = 7.5, 1.5 Hz), 2.47–2.37 (1H, m), 2.25–2.08 (5H, m), 1.94–1.83 (1H, m), 1.78–1.67 (1H, m) overlaying 1.69 (3H, s), 1.64–1.54 (1H, m), 1.46–1.36 (1H, m), 1.11 (3H, d, *J* = 7.0 Hz); δ_C (101 MHz, $CDCl_3$) 151.3, 144.9, 125.5, 124.6, 112.2, 108.6, 45.4, 45.2, 36.7, 35.9, 32.6, 32.5, 31.5, 20.7, 18.9.

^{1}H NMR (400 MHz, $CDCl_3$) [dr ~2.8:1]

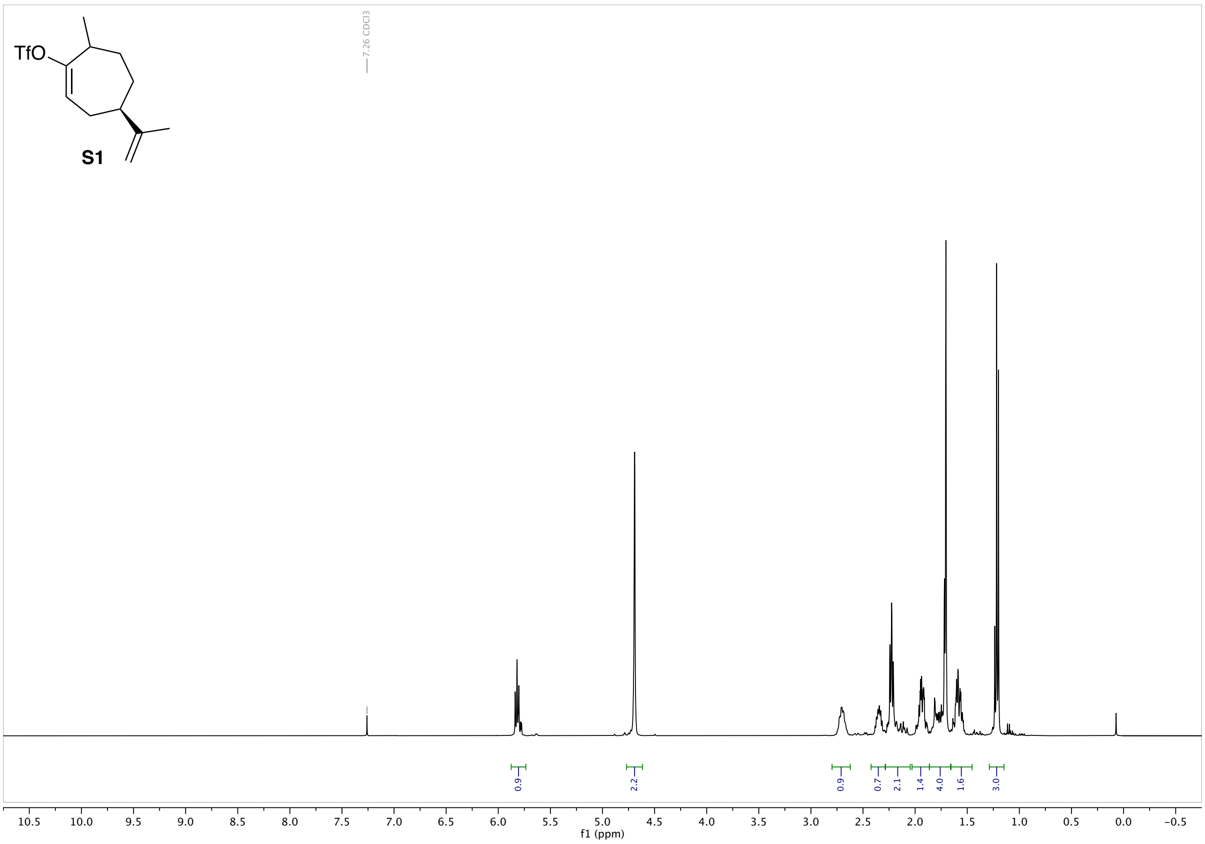

^{13}C NMR (101 MHz, $CDCl_3$) [dr ~2.8:1]

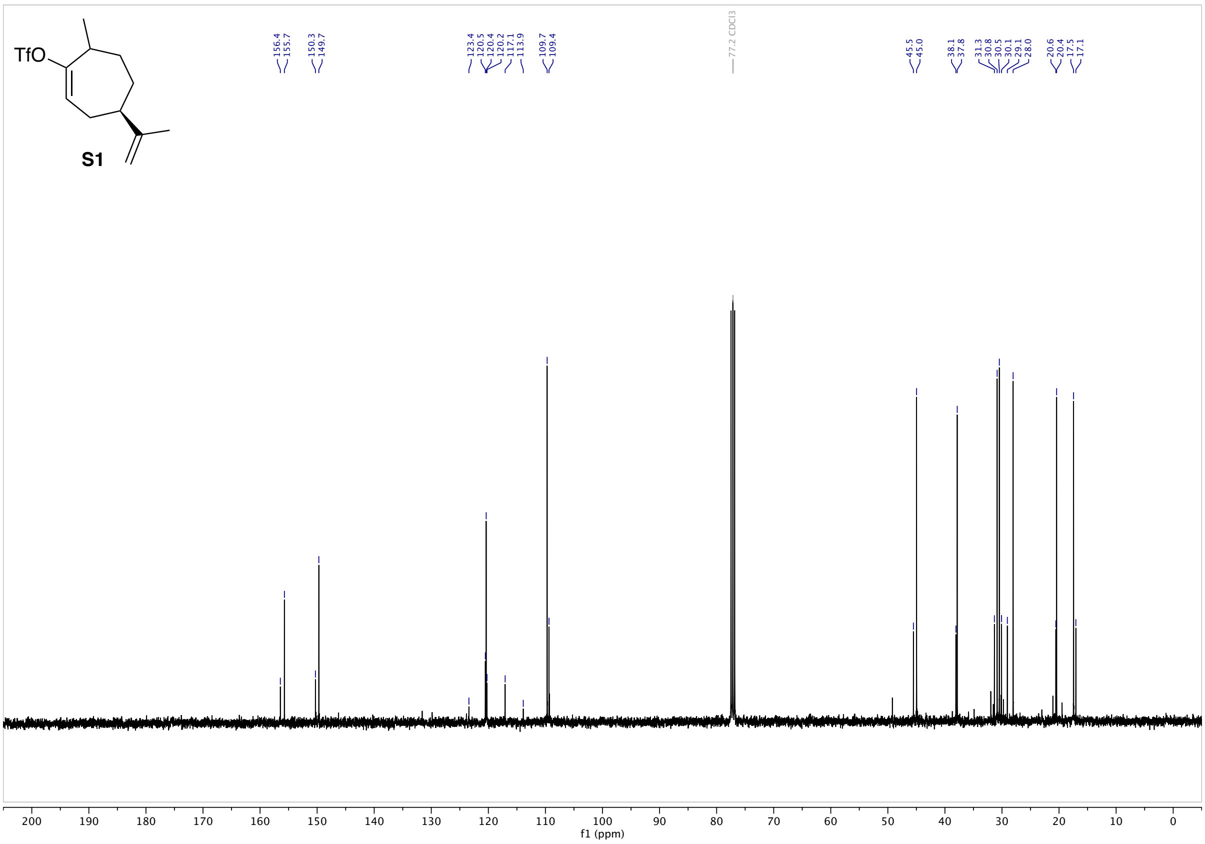

^{1}H NMR (400 MHz, $CDCl_3$) [contains BHT stabiliser]

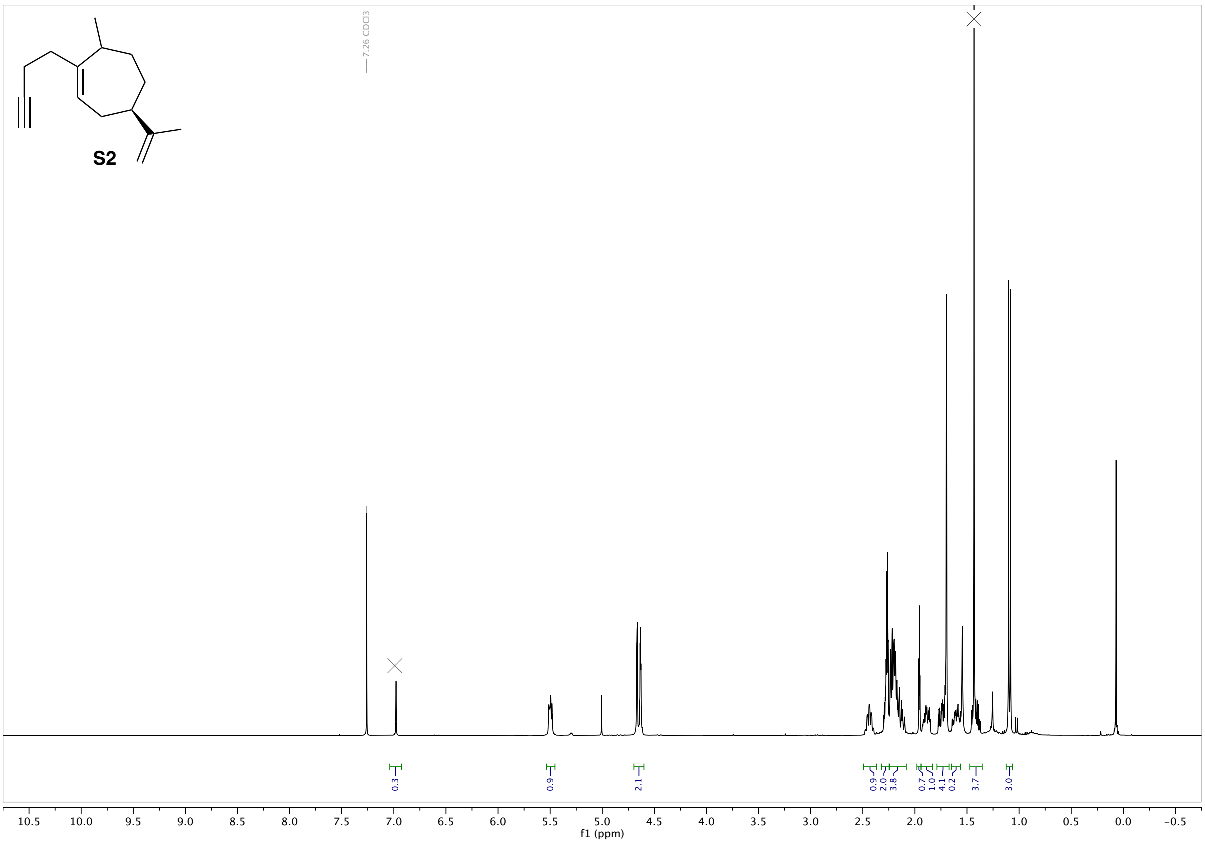

^{13}C NMR (101 MHz, $CDCl_3$)

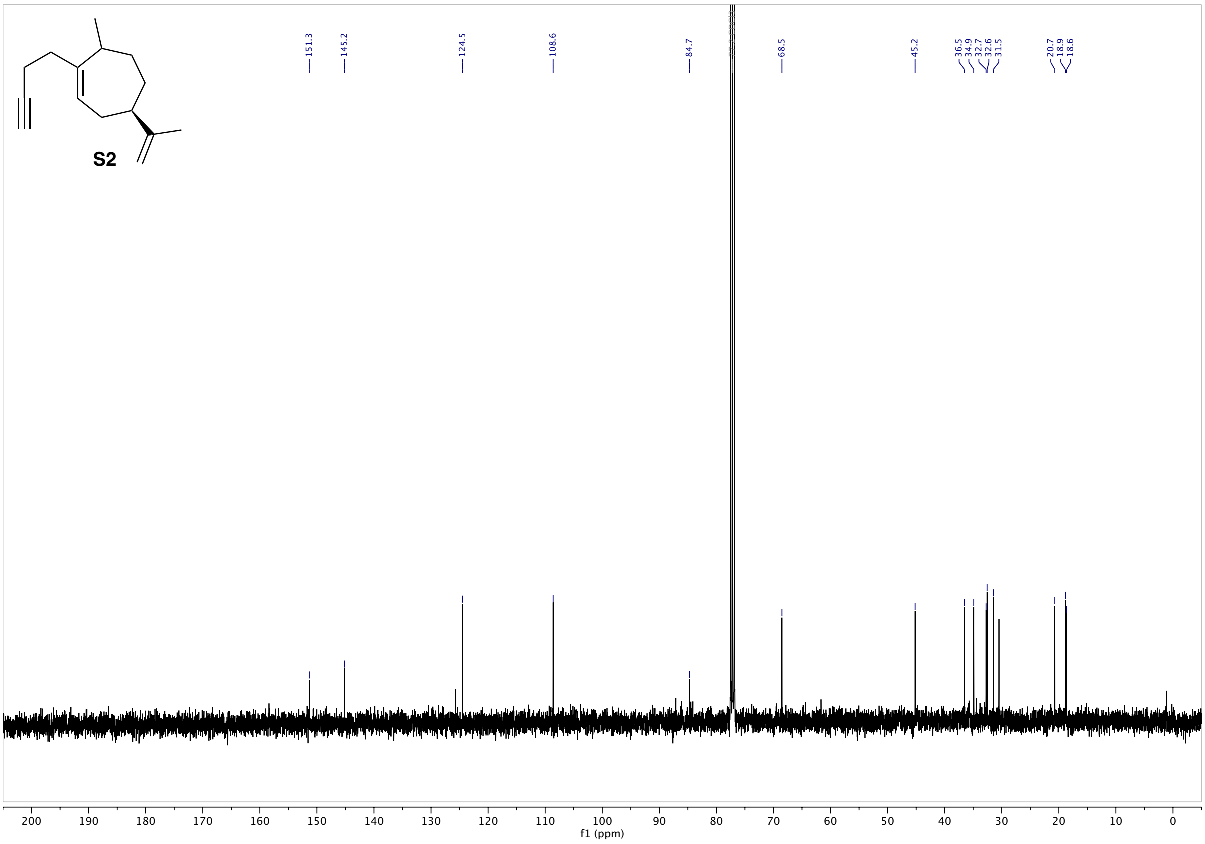

^{1}H NMR (400 MHz, $CDCl_3$)

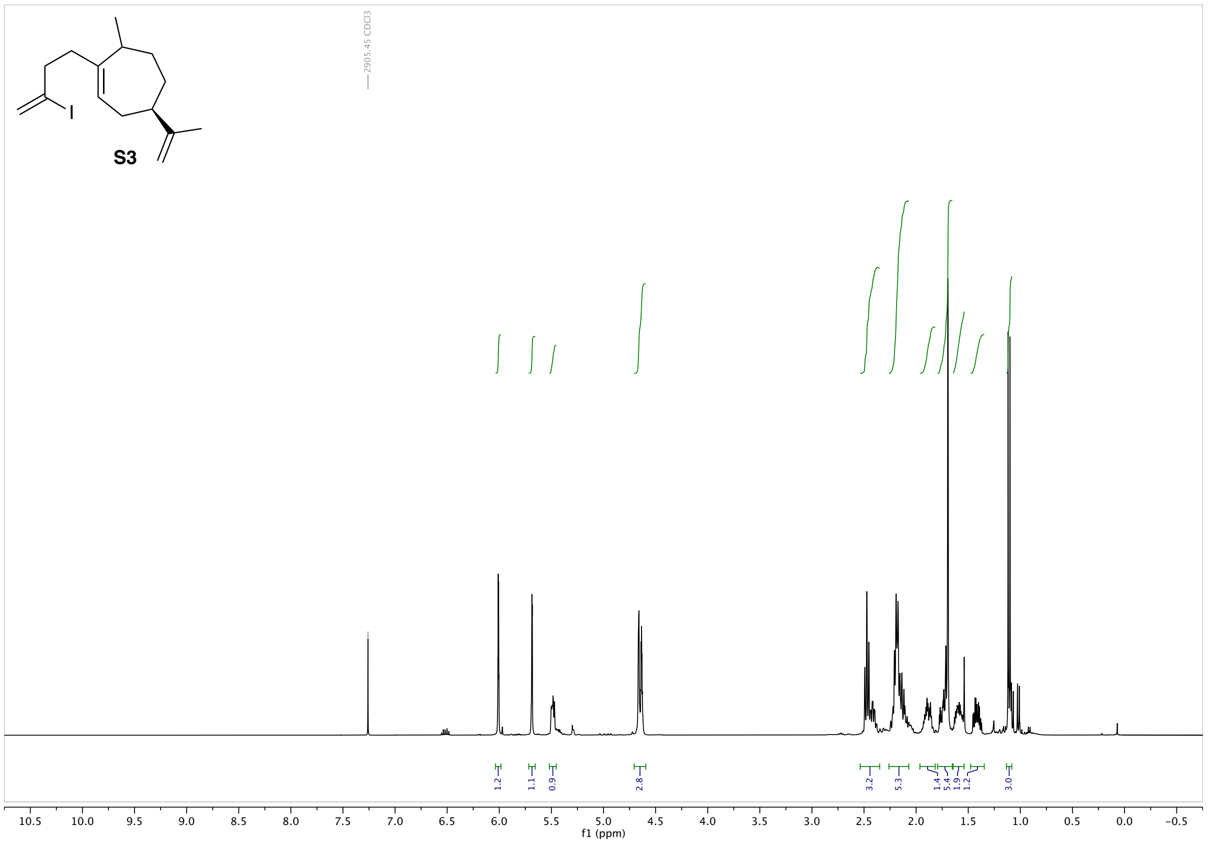

^{13}C NMR (101 MHz, $CDCl_3$)

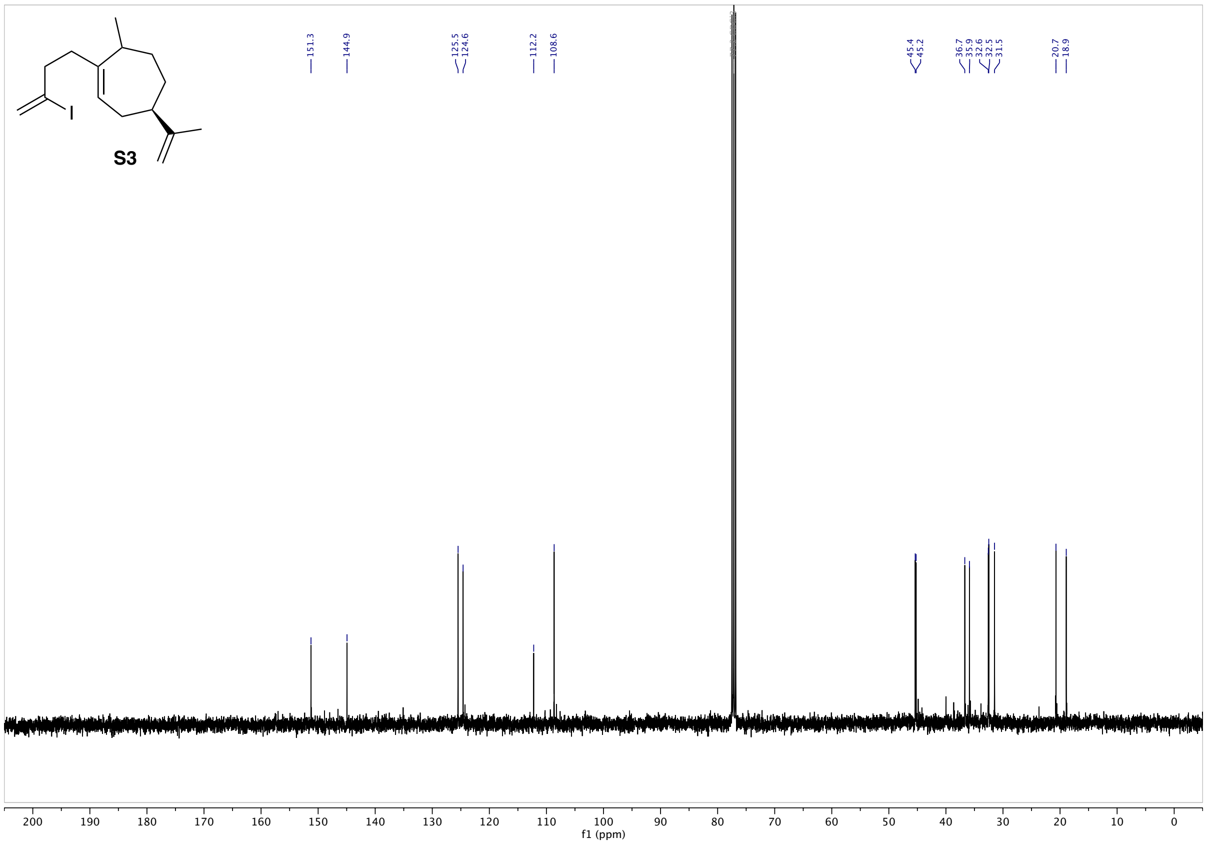

Optimisation of the butenylation reaction leading to ketone 18

Table S1. Direct butenylation of enone **14**.

Conditions trialled for the deprotonation of enone **14** and enolate alkylation with CH_2=$CHCH_2CH_2X$.

Entry	Base (eq.)	Additive (eq.)	Solvent	X (eq.)	T / °C	Time / h	Yield of 18
1	*t*-BuOK (1.2)	HMPA (30)	THF	I (1.5)	20	18	25
2	*t*-BuOK (1.1)	NaI (0.5)	THF	Br (1.2)	20	18	trace
3	NaH (2)	–	THF	I (2)	20 → 60	3	11
4	NaH (2)	–	THF	I (2)	66	48	trace
5	KHMDS (1.2)	HMPA (4.5)	THF	Br (1.3)	–78 → 20	18	trace
6	KHMDS (1.2)	–	THF	I (18)	–78 → 20	2	13
7	NaH (2), *t*-BuOK (1.2)	DMPU (10)	Toluene	Br (1.3)	20	2	trace

Table S2. Direct butenylation of enone **13**.

Conditions trialled for the deprotonation of enone **13** and enolate alkylation with CH_2=$CHCH_2CH_2X$.

Entry	Base (eq.)	Additive (eq.)	Solvent	X (eq.)	T / °C	Time / h	Yield of 18
1	*t*-BuOK (1.4)	–	THF	I (1.5)	20	18	25
2	*t*-BuOK (1.1)	DMPU (12)	DMF	I (1.3)	20	18	21
3	LHMDS (1.25)	–	THF	I (1.3)	20	18	trace
4	*t*-BuONa (1.2)	–	THF	I (1.3)	20	18	trace
5	KHMDS (1.2)	–	DMF	I (1.5)	20	18	trace
6	LHMDS (1.25)	–	DMF	I (1.5)	–30 → 20	18	trace
7	*t*-BuOK (1.2)	–	DMF	Br (1.3)	20	18	16
8	*t*-BuOK (1.2)	–	DMF	Br (1.3)	20 → 0[a]	2	10
9	*t*-BuOK (1.2)	–	*t*-BuOH	Br (1.3)	20	6	trace

[a] The base was added at 20 °C; after enolisation, the mixture was cooled to 0 °C and the electrophile added.

Table S3: Alkylation of enone **14** with 1,4-dihalobut-2-ene.

Conditions trialled for the deprotonation of enone **14** and enolate alkylation with (*E*)-XCH_2CH=$CHCH_2X$.

Entry	Base (eq.)	Additive (eq.)	Solvent	X (eq.)	T / °C	Time / h	Yield of 18
1	KHMDS (1.2)	–	THF	Cl (1.5)	–78 → 20	2	trace
2	KHMDS (1.2)	HMPA (4)	THF	Cl (1.3)	–78 → 20	2	25%
3	KHMDS (1.2)	–	THF	Br (5)	–78 → 20	18	mixture
4	KHMDS (1.2)	DMPU (10)	THF	Cl (2)	–78 → 20	6	trace
5	LDA (1.3)	DMPU (1.1)	THF	Cl (4)	–78 → 20	18	trace
6	NaH (2), *t*-BuOK (1.1)	–	Toluene	Br (1.5)	20	2	33%
7	NaH (2), *t*-BuOK (1.1)	–	Toluene	Br (4)	–78 → 20	2	73%

Table S4: Reductive rearrangement of ketone **15**.

Conditions trialled for the conversion of vinyl cyclopropane derivative **15** into **18**, minimising the co-production of vinyldihydrofuran derivative **17**.

Entry	Pd_2dba_3 (mol%)	Bu_3P (mol%)	Reducing agent (eq.)	T / °C	Ratio 18:17 (NMR)	Yield of 18
1	10	80	HCO_2NH_4 (2.2)	105	100:0	31 (mixture)
2	2	–	HCO_2NH_4 (2.2)	105	–	0
3	2	16	HCO_2NH_4 (2.2)	105	17:83	(not isolated)
4	2	16	Et_2Zn (3)	75	0:100	0
5	2	16	HCO_2NH_4 (2.2) + H_2O (2)	105	27:73	(not isolated)
6	2	16	HCO_2NH_4 (2.2) + H_2O (55)	105	27:73	(not isolated)
7	2	16	HCO_2H (5) + HCO_2NH_4 (2.2)	105	50:50	(not isolated)
8	2	16	HCO_2H (5) + Et_3N (2)	105	80:20	(not isolated)
9	2	16	HCO_2H (7) + Et_3N (2)	105	80:20	51
10	2	16	HCO_2H (14) + Et_3N (4)	105	100:0	89
